# The Role of Attitude Strength in Behavioral Spillover: Attitude Matters—But Not Necessarily as a Moderator

**DOI:** 10.3389/fpsyg.2019.01018

**Published:** 2019-05-09

**Authors:** Adrian Brügger, Bettina Höchli

**Affiliations:** Department of Consumer Behavior, Faculty of Business, Economics and Social Sciences, University of Bern, Bern, Switzerland

**Keywords:** pro-environmental behavior, health behavior, environmental attitude, health attitude, spillover, moral licensing, moral cleansing

## Abstract

Studies on how one behavior affects subsequent behaviors find evidence for two opposite trends: Sometimes a first behavior increases the likelihood of engaging in additional behaviors that contribute to the same goal (positive behavioral spillover), and at other times a first behavior decreases this likelihood (negative spillover). A factor that may explain both patterns is attitude strength. A stronger (more favorable) attitude toward an issue may make the connections between related behaviors more salient and increase the motivation to work toward the underlying goal. We predicted that people with a stronger (more favorable) attitude are more likely to engage in subsequent behaviors that address an issue they care about. Two experiments tested the prediction in the contexts of pro-environmental and health behavior. Study 1 (*N* = 378) provided some support for the predicted moderating role of attitude toward the environment when participants recalled either an environmentally friendly or unfriendly action: A strong attitude increased the likelihood, whereas a weak attitude decreased the likelihood of carrying out successive goal-conducive behaviors. When compared to a neutral control condition in Study 2 (*N* = 929), participants with a weak environmental attitude supported pro-environmental petitions *less* strongly after an environmentally harmful action. Support for such petitions did not waver, however, among participants with a strong environmental attitude: They consistently acted environmentally friendly. Contrary to the hypothesis, in neither study did strength of attitude toward personal health moderate the effect of an initial behavior in the expected direction. In sum, the two studies provided only limited evidence for behavioral spillover: Participants mostly acted in accordance with their attitude but were hardly affected by recalling previous actions. When behavioral spillover did occur, however, a strong environmental attitude tended to increase the likelihood of acting in an environmentally friendly way, whereas the behavior of those with a weak attitude was less predictable. This research contributes to a more nuanced theoretical understanding of the role of attitude in spillover, but provides only limited evidence for its role as a moderator.

## Introduction

Many personal and societal goals can be achieved only if people repeatedly work toward them. For example, to lead a healthy life, it is not enough to eat a single healthy meal. People need to repeatedly make healthy food choices and also do other things that benefit their health, like get enough sleep and exercise regularly. Similarly, if people want to reduce their environmental footprint, they need to do more than recycle one glass bottle; they need to repeatedly recycle different types of things and engage in additional behaviors, such as using energy-efficient appliances and modes of transport. In short, in many contexts people need to engage in several successive actions to achieve their goals.

**FIGURE 1:**
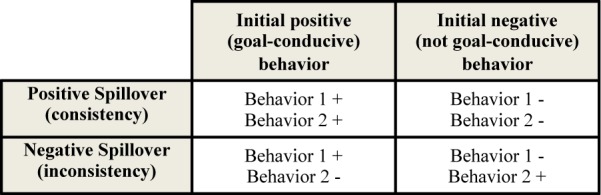
Overview of how the valence and (in)consistency of successive behaviors lead to positive and negative spillover (adopted from Dolan and Galizzi, 2015).

Despite the need for such consistent behavior, we know relatively little about when an action that helps achieve a goal affects subsequent actions that contribute to the same goal. In accordance with previous research, we refer to relationships between initial and subsequent behaviors as “spillover.” *Positive* spillover refers to situations where a first behavior increases the likelihood of a different second behavior (i.e., spillover across behaviors), or the same behavior again across time (i.e., spillover across time) or in a different context (i.e., spillover across contexts) that contributes to the same goal as the first (Truelove et al., 2014; Dolan and Galizzi, 2015; Nilsson et al., 2017; Carrico et al., 2018). By contrast, *negative* spillover describes situations in which a first goal-conducive behavior *reduces* the likelihood of engaging in other, similar behaviors or the same behavior across time or contexts (or in which a first, goal-inconsistent behavior increases this likelihood, see Figure 1 for all the variations).

The literature provides compelling theoretical explanations and empirical evidence for both types of spillover (Dolan and Galizzi, 2015). On the one hand, research in the context of moral behavior shows that after performing a first moral behavior, individuals feel that they have earned the moral entitlement to reward themselves by refraining from further moral behavior (Monin and Miller, 2001; Merritt et al., 2010). To illustrate, individuals who recalled a moral behavior were more likely to cheat on a math task (Jordan et al., 2011) and donated less money to charity (Sachdeva et al., 2009). Other research corroborates the idea that an initial behavior can induce the feeling that a person has “done enough” and that no further behavior along the same lines is necessary, which fosters negative spillover effects (variously termed resting on one’s laurels, Amir and Ariely, 2008; goal attainment, Longoni et al., 2014; single-action bias, Weber, 1997a).

On the other hand, other perspectives such as cognitive dissonance theory (Festinger, 1957), self-perception theory (Bem, 1972), and the foot-in-the-door effect (Freedman and Fraser, 1966) suggest that individuals have a strong urge for consistency and tend to act in a way that is consistent with previous actions and existing beliefs, which should lead to positive spillover (Albarracín and Wyer, 2000; Gawronski and Strack, 2012).

A crucial question that arises from these two contradictory patterns of spillover concerns why a first goal-conducive behavior sometimes increases the likelihood of further similar behaviors and why it sometimes reduces it. One explanation is that additional psychological processes may be at work (Truelove et al., 2014; Mullen and Monin, 2016). For example, it is possible that the extent to which a behavior and its broader context matter to a person influences which psychological processes are triggered and whether they result in positive or negative spillover (Effron et al., 2009; Meijers, 2014; Nilsson et al., 2017). Our research builds on this idea: We argue that the more a person cares about an issue such as the environment or personal health – the strength of their attitude – the more likely they are to engage in multiple behaviors conducive to the underlying goal (positive spillover). By contrast, when people engage in behaviors to do with issues they do not care strongly about, they feel they have done enough (Weber, 1997b; Amir and Ariely, 2008), and use their limited resources (e.g., attention, physical strength, time, money) to pursue other goals (Moskowitz, 2012).

Previous spillover research focused on behaviors with obvious links to morality, and often relied on moral processes to explain spillover effects (including behaviors connected to environmental protection, which has clear moral connotations; Monin and Miller, 2001; Effron et al., 2009; Mazar and Zhong, 2010; Merritt et al., 2010; Meijers, 2014). We tie in to this research tradition by using an established experimental paradigm (Sachdeva et al., 2009), examining the predicted moderating influence of attitude strength on spillover in the context of environmental protection, which is often strongly morally connoted (e.g., Feinberg and Willer, 2013). We extend the scope of previous research by testing assumptions in two different contexts: environmental protection and health. As a result, we explore whether spillover processes are restricted to behaviors related to morality or whether they also occur in domains less morally charged.

### Personal Relevance as a Moderator of Behavioral Spillover

The idea that personal relevance could influence the extent and type of behavioral spillover is supported by different theoretical perspectives and some empirical evidence. We take a goal-theoretical perspective to reconcile different streams of research into conceptually similar constructs (e.g., superordinate goals or identity). The central hypothesis is that the more relevant an issue is to a person, the more an initial goal-conducive act should decrease negative spillover and promote positive spillover (see Höchli et al., 2018).

According to goal-theoretical perspectives, people pursue goals that are related to each other but vary in level of abstraction (Vallacher and Wegner, 1987; Carver and Scheier, 2001). For example, “be healthy” is a relatively abstract and broad health goal at the top of the hierarchy, whereas “do 40 push-ups on Wednesday afternoon” is a specific health goal at the bottom (Carver and Scheier, 2001; Kruglanski et al., 2002). The most concrete goals (sub-goals) correspond to specific, single actions.

More abstract goals are often referred to as “superordinate” (Carver and Scheier, 2001). These broad representations determine what people ultimately value and aspire to; they provide a general orientation as to what is important to a person (Carver and Scheier, 2001; Schwartz et al., 2001; Boekaerts et al., 2006).

This understanding of superordinate goals points to similarities with functionally and conceptually related concepts. For instance, goals are often equated with values (e.g., Schwartz, 1992). Further, superordinate goals are described as “be” goals – that is, the kind of self one aspires to be (Carver and Scheier, 2001). This links superordinate goals closely to theoretical concepts such as “self-identity” and “possible selves,” which are as well representations of the self that motivate behavior (Hoyle and Sherrill, 2006; Oyserman and James, 2011; Van der Werff et al., 2013). Although superordinate goals, values, identity, and possible selves are theoretically distinct concepts, the terms are often used interchangeably (Schwartz, 1992; Masuda et al., 2010).

There are at least two characteristics of superordinate goals that point to their possible role as moderators of spillover. First, the intrinsic importance of superordinate goals and their crucial role for the overriding sense of self (Carver and Scheier, 2001) can have a stabilizing effect on behavior. More specifically, it is likely that people experience cognitive dissonance if they engage in behaviors that jeopardize their superordinate goals (Festinger, 1957). Because cognitive dissonance is unpleasant, avoiding it could be an important driver for consistently carrying out goal-conducive behaviors (Sintov et al., 2019). Similar arguments can be made concerning theories of identity and self-perception: The more people see themselves as environmentalists or health-conscious persons, the more they are likely to experience cognitive dissonance and negative emotions such as guilt or remorse when they do not act according to their identity or self-perception (Lanzini and Thøgersen, 2014; Van der Werff et al., 2014a; Byrka and Kaminska, 2015; Lacasse, 2016). Importantly, this stabilizing effect can be expected only among people who hold relevant superordinate goals. This is why we expect superordinate goals to moderate spillover: To the extent that people hold a superordinate goal (or have strong values, identity, self-perception) in a given domain, the more they should engage in behaviors that qualify as positive spillover after an initial goal-conducive act (and as negative spillover after an initial act that is inconsistent with their goal) (Fishbach et al., 2006; Thøgersen and Crompton, 2009; Meijers et al., 2014; Nilsson et al., 2017).

Second, the interconnected structure of goals is likely to enhance this stabilizing effect. Superordinate goals typically include multiple concrete sub-goals that are instrumental to achieving them (Carver and Scheier, 2001; Kruglanski et al., 2002). For example, to “be healthy,” a person needs to do more than hit the gym once a week – they need to be physically active in other ways as well (e.g., take the stairs instead of the elevator), and pursue additional broad and specific health goals such as “eat healthily” and “have fruit instead of a chocolate bar as a snack.” It can be assumed that the more people represent an issue as a superordinate goal (i.e., the more it matters to them), the more salient are the connections between the superordinate goal and relevant behaviors, and the more different goal-conducive behaviors should be linked to each other through the superordinate goal. A characteristic of this interconnectedness is that goals can activate (or inhibit) each other: Dealing with a concrete action or a subordinate goal can activate the associated superordinate goal (bottom-up activation; Shah and Kruglanski, 2003), and focusing on a superordinate goal can activate the associated subordinate goals or actions (top-down activation; Kruglanski et al., 2002). Thus, when people carry out a behavior for which they have a corresponding superordinate goal, this should increase the salience of the goal, highlight the importance of carrying out other goal-conducive behaviors, and increase the likelihood of doing so (Bargh et al., 1992; Ratneshwar et al., 2001; Kruglanski et al., 2002; Thøgersen and Noblet, 2012). Positive spillover effects can therefore be understood as the result of an initial goal-conducive behavior that activates a superordinate goal, that in turn guides other behaviors (Lanzini and Thøgersen, 2014; Margetts and Kashima, 2017). Again, this process is contingent on people holding a relevant superordinate goal (or identity, self-perception, values).

Support for this idea comes, for example, from a community field experiment that tested an intervention to save electricity (Steinhorst et al., 2015). Participants received electricity-saving tips, combined with either a monetary (savings in euros) or an environmental framing (savings in CO_2_), or no framing in the control group. Although an increase in the target behavior – saving electricity – was observed in both framing groups, spillover to other pro-environmental behaviors was observed only in the environmental condition.

There is also empirical evidence to support the idea that the more importance people attach to an issue or a cause, the more they tend to engage in behaviors that maintain, advance, and defend it. To illustrate, the effect of personal importance on behavior is evident in positive correlations between a broad range of environmentally friendly behaviors and concepts related to the personal importance of environmentalism, such as an *ecocentric belief structure* (i.e., humans are a part of natural systems and constrained by their limits; Dunlap and Van Liere, 1978; see also Olli et al., 2001; Kortenkamp and Moore, 2006), *self-transcending* and *biospheric* *values* (Karp, 1996; Stern et al., 1998; Schultz, 2001; Schultz et al., 2005; Thøgersen and Ölander, 2006; Gatersleben et al., 2014), *connectedness to nature* (Schultz, 2001; Brügger et al., 2011; Otto and Pensini, 2017), *identity/self-perception* as someone who acts in an environmentally friendly way (Nigbur et al., 2010; Whitmarsh and O’Neill, 2010; Gatersleben et al., 2014; Kashima et al., 2014; Van der Werff et al., 2014b; Meijers et al., 2015), and *environmental* *attitude* (Hines et al., 1986; Bamberg and Möser, 2007). Similar relationships can also be found between higher scores on similar concepts and health behavior (e.g., Theodorakis, 1994; Godin and Kok, 1996; Sparks and Guthrie, 1998; Hagger et al., 2007).

The literature also holds more direct evidence for the idea that following an initial goal-conducive act, personal importance should increase positive and reduce negative spillover. For instance, the higher people score on measures that reflect personal importance, the less likely they are to endorse the idea that they can justify or neutralize environmentally harmful behaviors with other, more environmentally friendly behaviors (Bratt, 1999; Kaklamanou et al., 2015).

The most direct support for the idea that personal importance can explain behavioral spillover comes from three experiments that examined how a first behavior affected a second behavior. The first study found that the expression of a non-racist intention (to vote for Obama in the 2008 election) tends to lead to racist behavior (allocating more resources to Whites than Blacks), but only for those with higher racist scores (Effron et al., 2009, Study 3).

Another study found that after imagining purchasing an environmentally friendly product, participants with a strong environmental identity tended to express pro-environmental intentions to the same extent as their counterparts who had bought a conventional product. By contrast, when participants with a weak environmental identity purchased an environmentally friendly product, they expressed lower environmentally friendly intentions than after buying the conventional product (Meijers, 2014).

The third experiment (Noblet and McCoy, 2018) manipulated whether participants perceived their past ecological behavior as either environmentally friendly or unfriendly, then asked them how strongly they supported a pro-environment energy policy. It was found that the perception of one’s past behavior as environmentally friendly decreased support for the policy among those with low intrinsic environmental motivation. However, those with high environmental motivation supported the policy to an equal extent, irrespective of whether they were led to see their past behavior as environmentally friendly or not. These studies provide compelling initial evidence for the idea that after an initial goal-conducive behavior, personal importance – in the reported studies, operationalized as attitude, identity, or intrinsic environmental motivation – leads to positive spillover effects, whereas low personal importance leads to negative spillover effects.

### Behavior-Based Attitude as a Measure of Personal Importance

From a methodological point of view, how to measure abstract concepts such as personal relevance, superordinate goals, values, or possible selves is not a trivial matter. It is *technically* feasible to ask questions that directly tap into such abstract concepts: Schwartz (1992) assessed values by asking people to indicate the extent to which different values act as “guiding principles” in their lives. However, such direct ways of assessing abstract concepts require introspection and self-reflection. This is problematic because abstract concepts are by definition difficult to grasp intellectually; respondents may not necessarily understand the concepts in the same way researchers do. A second problem is that the information required to evaluate such abstract concepts is often not readily available, which makes these types of question prone to recollection bias (Dillman, 2001), response bias (e.g., Wittenbrink and Schwarz, 2007), and social desirability bias (Crowne and Marlowe, 1960).

In this paper, we take an *indirect* approach to measuring personal relevance that is grounded in the Campbell paradigm (Kaiser et al., 2010), an innovative paradigm from attitude research. Based on Donald Campbell’s conceptualization of attitude as an “acquired behavioral disposition” (Campbell, 1963, p. 97), Kaiser et al. (2010) argue that attitudes and behaviors are formally – but not causally – linked. This means that a latent attitude is manifest in people’s behaviors and, conversely, that the attitude denotes the subjective importance of the behavior to the person (Kaiser et al., 2010). A second crucial proposition of Kaiser et al. (2010) is that behavior is determined by two factors: (1) the strength of the latent attitude and (2) the costs of the behavior (e.g., money, physical effort, time, sacrifice, or social risk).

An implication of this conceptualization is that the latent attitude can be inferred from a *systematic* inspection of behaviors that are ordered according to their cost (Kaiser et al., 2010): The more costly, difficult, and demanding a person’s behaviors are, the stronger must be their corresponding attitude. Why would someone install expensive solar panels or spend a lot of time traveling by train rather than by airplane if they did not have a strong environmental attitude? Likewise, when the tiniest difficulty is enough to stop a person from engaging in a healthy behavior, their health attitude is probably weak.^1^

Conceptualizing attitude as a behavior-based latent trait has several advantages: Answering questions about past actions requires a minimal amount of introspection (see Otto et al., 2018). Therefore, answering questions about one’s behavior should be easier than answering questions about abstract concepts such as superordinate goals, values, or identity. Furthermore, previous research suggests that questions about one’s behavior are less vulnerable to response biases such as social desirability than conventional attitude questions (Milfont, 2009). Moreover, behavior-derived attitudes are relatively stable across time (Kaiser et al., 2014), which makes them particularly useful for measuring trait-like individual preferences.

This approach of assessing latent constructs through behaviors has already been implemented in various contexts. They include environmental attitude (Kaiser et al., 2013, 2014; Ogunbode et al., 2018), attitude toward nature (Brügger et al., 2011; Kaiser et al., 2013, 2014), attitude toward climate change (Urban, 2016), health attitude (Byrka and Kaiser, 2013), attitude toward conformity (Brügger et al., 2019), and need for recovery at work (Smolders et al., 2012). Although most instruments developed within the Campbell paradigm are formally denoted as attitude scales, the latent trait being assessed can also be thought of as an indication of people’s motivation: how “personally important” a goal is to them (Kaiser et al., 2017). As such, using behavior-based attitude scales is a promising approach to measuring the extent to which environmental protection and health are personally important to people.

### Overview of Studies

The goal of the research is to examine whether personal importance – operationalized as the strength of behavior-based attitude – can shed light on when positive and negative behavioral spillover occurs. To examine the role of attitude strength as a moderator, we conducted two experiments. In both, we used an experimental paradigm that is often used in research on moral licensing (Blanken et al., 2015): Participants recalled a recent past behavior that was either consistent or inconsistent with the goal to be healthy or to protect the environment, and that therefore had the potential to trigger spillover effects, and then answered questions about future behaviors.

Using this recall paradigm offers at least three advantages over other approaches. First, participants are not forced to carry out behaviors that they would not do of their own free will, which could otherwise raise ethical questions for researchers. Second, using a design in which participants are either selected because they already perform a specific behavior or are asked to adopt a specific behavior could lead to samples in which, for example, relevant individual attitudes are already very positive. Using the recall paradigm should result in more inclusive samples in which the variance in participants’ attitudes is not restricted. Third, asking participants to describe an event of their own choice guarantees that the behavior has the intended subjective meaning (see also Thøgersen, 2004).

Study 1 provided initial evidence for the expected role of attitude strength as a moderator. However, it did not include a neutral control group and its sample (*N* = 378) consisted mainly of female students. By using a broader and larger sample (*N* = 929) and by including an additional neutral condition, Study 2 overcame these shortcomings, and again found some support for the predicted role of attitude strength as a moderator.

## Study 1

To examine the moderating influence of attitude strength, we tested for interaction effects between the experimental conditions (recalling a behavior that was consistent vs. inconsistent with the goals to protect the environment and to be healthy) and attitude strength in the contexts of environmental protection and health. (For a similar approach, see Conway and Peetz, 2012; Cornelissen et al., 2013; Noblet and McCoy, 2018.)

We predicted that participants with a strong attitude would engage in positive spillover after an initial goal-conducive behavior and in negative spillover after an initial goal-inconsistent behavior, leading to high motivation to engage in goal-conducive behaviors in both experimental conditions. These predictions were based on the following assumptions: When participants with a strong attitude carry out a behavior that is relevant to their attitude, this should (a) increase the salience of their attitude; and (b) the relationships between different attitude-relevant behaviors and how they are relevant to the underlying attitude; and (c) they would experience cognitive dissonance if behaviors were inconsistent with their attitude.

By contrast, we expected that, after recalling a goal-consistent behavior, participants with low attitude strength would feel that they had “done enough” and therefore be less motivated to engage in further behaviors than their counterparts who recalled a goal-inconsistent behavior.

### Materials and Methods

#### Procedure

Data were collected through a web-based survey tool (Qualtrics) in spring 2013.

To reduce the risk that questions about participants’ attitudes had carryover effects on either the recall manipulation or the dependent variables, we collected the data at two points in time. At time 1, respondents were asked if they wanted to participate seriously or only look at the survey. A “seriousness check” is a recommended means of reducing dropout rates and increasing data quality (Reips, 2002). Participants then answered questions about their attitudes toward the environment, health, and various risks. These items were intermixed and presented in eight question blocks. The risk-related questions were filler items. The survey also included socio-demographic questions.

At time 2 (10–14 days later), participants were again asked if they were willing to participate seriously. They then completed one of four recall conditions, to which they were assigned randomly. After a short filler task (unscramble 12 sequences of four to eight letters into words), participants answered the questions that were used as dependent variables. Finally, participants completed a manipulation check, were thanked and debriefed.

#### Participants

The sample was recruited via various Swiss Internet forums (e.g., Swiss variations of Craigslist such as pinwand.ch, platforms for students such as students.ch) and social media networks. As an incentive, those who participated in both parts of the survey were entered in a raffle to win Amazon vouchers (4 × EUR 100 and 10 × EUR 10). In total, 738 participants accessed the survey at time 1. Of those, 190 were removed because they responded to fewer than 20% of the questions or because they participated more than once (in which case we discarded the second participation). Of the 548 participants who participated at time 1, 490 accessed the study at time 2. Two participants participated twice; we again excluded the answers from their second participation.

To ensure good data quality, we retained participants only (a) who in both parts passed the seriousness check (Reips, 2002), (b) whose participation time in both surveys lasted at least one third of the sample’s median time (16 min at time 1; 17 min at time 2), and (c) who provided a semantically meaningful answer in the recall task (judged by two independent raters). The mean age of participants who met these criteria (*N* = 378) was 28.78 (*SD* = 9.29). The proportion of women was 71%. Of the participants who revealed their academic affiliation, 61% were students.

A comparison between the 170 participants who participated at time 1 but either did not participate at time 2 or did participate but were excluded to ensure good data quality and the 378 participants who were retained for the analyses revealed that the proportion of these two groups was not associated with the experimental conditions [χ^2^(3) = 0.45, *p* = 0.93]. However, the 378 participants who were retained had a more environmentally friendly attitude (*M* = 0.12, *SD* = 0.85) than those excluded [*M* = -0.10, *SD* = 0.96; *t*(294.18) = -2.56, *p* = 0.01]. Importantly, though, this self-selection bias did not reduce the variance in environmental attitude, which suggests that the sample was still broad enough to conduct the intended analyses. The two groups did not differ with respect to health attitude, *t*(324.98) = -1.57, *p* = 0.12.

#### Manipulation

Participants were randomly assigned to one of four experimental conditions in which they were asked to recall one of the following types of behavior carried out during the past week: (1) environmentally friendly, (2) environmentally harmful, (3) healthy, or (4) unhealthy. Participants were instructed to take 5–10 min to write down their action in detail (Jordan et al., 2011; Weibel et al., 2014).

To examine whether the manipulation had the intended effect, two manipulation checks were used. First, participants were asked to indicate the valence of the described deed (seven-point scale: -3 = very negative, +3 = very positive). Second, two coders who were blind to conditions rated how environmentally friendly and healthy the deeds were (seven-point scale: -3 = very environmentally harmful/very unhealthy, +3 = very environmentally friendly/very healthy) (Jordan et al., 2011). Interrater reliability was high for both contexts (intraclass correlation coefficient [ICC]_environmentallyfriendly_] = 0.92, ICC_healthy_ = 0.93). The ratings of the two coders were combined to create an environmental friendliness and a healthiness scale.

#### Moderators

To test the hypothesis that the extent of positive and negative spillover is contingent on people’s attitudes, we included two behavior-based attitude scales (Kaiser and Wilson, 2004; Byrka and Kaiser, 2013; Kaiser et al., 2014). Following Kaiser et al.’s (2010) suggestion, we used the probabilistic Rasch model (for details, see Bond and Fox, 2007) to estimate attitude levels for persons and behavioral difficulties. This approach is consistent with previous implementations of the Campbell paradigm (Smolders et al., 2012; Kaiser et al., 2013; Urban, 2016; Ogunbode et al., 2018; Brügger et al., 2019).

*Environmental attitude* was measured with 50 items from Kaiser and Wilson (2004) (see Supplementary Table 1). Of the 50, items 32 were presented in a five-point frequency format. Responses to these items were recoded into a dichotomous format by collapsing “never,” “seldom,” and “occasionally” into “unreliable pro-environmental engagement,” and “often” and “always” into “reliable pro-environmental engagement.” The remaining 18 items were presented in a yes/no format. Nineteen behaviors represented environmentally unfriendly activities and were recoded prior to analysis. The dichotomization, calibration of the behavior scale, and estimation of person scores were based on the classical Rasch model and consistent with previous calibrations of the same instrument (see Kaiser and Wilson, 2004). Attitude scores were estimated in logits; the more negative the score, the weaker the person’s environmental attitude. All behavior items were found to fit the model very well (infit mean square values < 1.18; for reference values, see Bond and Fox, 2007). The Rasch-model-based reliability estimate of the measure was *rel* = 0.80.

*Health attitude* was measured with 46 items from Byrka and Kaiser (2013) and five items from Kibbe (2011) (Supplementary Table 2). For 27 items, we used a five-point frequency answer scale and then dichotomized responses in a similar way as for the environmental scale. The remaining 24 items were presented in a yes/no format. Nine items represented unhealthy behaviors and were recoded prior to analysis. The dichotomization, calibration of the behavior scale, and estimation of person scores were again based on the classical Rasch model and consistent with previous calibrations (Byrka and Kaiser, 2013). All behavior items were found to fit the model very well (infit mean square values < 1.15). The Rasch-model-based reliability estimate of the measure was *rel* = 0.66.

#### Dependent Variables

To assess the extent of positive and negative spillover, we used two types of dependent variables as proxies for future goal-conducive behaviors. First, participants indicated on a seven-point scale (1 = I will not do that under any circumstances, 7 = I will certainly do that) the extent to which they intended to engage in 18 behaviors in different contexts during the next month. Of these *behavioral intentions*, five were related to protecting the environment and five concerned their personal health and were used as dependent variables (Table 1). The other eight were fillers.

**Table 1:** Descriptive statistics for behavioral intentions (I1–I5) and interest in apps (A1–A3) in the contexts of environment and health, Study 1.

	*Mean*	*Median*	*SD*	*Range*
**Environmental protection**				
I1: Composting green waste	4.71	6	2.35	1–7
I2: Using biodegradable cleaning agents	4.56	5	1.84	1–7
I3: Switching off electronic devices on standby completely overnight	4.93	5	1.90	1–7
I4: Buying locally grown vegetables and fruits	5.86	6	1.28	1–7
I5: Switching off lights when leaving a room	6.54	7	0.84	2–7
A1: Saving energy at work	4.38	4	1.78	1–7
A2: Saving energy at home	5.28	6	1.58	1–7
A3: How to reduce my CO_2_ emissions	4.71	5	1.79	1–7
**Health**				
I1: Treating myself with a high-calorie or fatty snack (e.g., chocolate bar or potato chips) (reverse-coded)	2.32	2	1.64	1–7
I2: Taking time to relax	5.51	6	1.42	1–7
I3: Exercising for at least 2 h per week	5.74	7	1.70	1–7
I4: Drinking no more than one glass of alcohol per day	4.62	5	2.24	1–7
I5: Preparing at least one fresh meal per day	5.55	6	1.59	1–7
A1: How to maintain a healthy diet	5.71	6	1.45	1–7
A2: Simple relaxation techniques in your spare moments	5.02	5	1.58	1–7
A3: More physical activity in everyday life	5.28	6	1.73	1–7


Second, we asked participants if they would be interested in using online apps that provided support and tips to better achieve goals. Of the nine apps, three were related to environmental protection and three to improving health (Table 1); the other three were fillers. Participants used a seven-point scale to indicate how much they were interested in these apps (1 = not interested at all, 7 = very interested).

### Results

#### Levels of Environmental and Health Attitudes in the Four Experimental Conditions

We first established that the random allocation of participants to the four conditions was successful with respect to the strength of attitudes. Levels of environmental [*F*(1,376) = 0.03, *p* = 0.86, η^2^ = 0.00] and health attitude [*F*(1,376) = 0.40, *p* = 0.53, η^2^ = 0.00] were not statistically different in the four conditions.

#### Manipulation Checks

##### Environmental behavior

Manipulation checks showed that the recall manipulation had the intended effect. Participants in the environmentally friendly condition rated the recalled environmental action as more positive (*M* = 5.63, *SD* = 0.99) than participants in the environmentally unfriendly condition (*M* = 3.10, *SD* = 1.14), *t*(179) = 16.04, *p* < 0.001, *d* = 2.39. Coders also rated the recalled environmental behaviors in the environmentally friendly condition as more positive (*M* = 2.00, *SD* = 0.61) than those in the environmentally unfriendly condition (*M* = -1.58, *SD* = 0.85), *t*(183) = 32.93, *p* ≤ 0.001, *d* = 4.84.

##### Health behavior

The recall manipulation had the intended effect. Participants in the healthy condition rated the recalled health behavior as more positive (*M* = 6.18, *SD* = 0.77) than participants in the unhealthy condition (*M* = 2.90, *SD* = 1.11), *t*(188) = 23.84, *p* < 0.001, *d* = 3.46. Coders rated the health behaviors in the healthy condition as more positive (*M* = 2.14, *SD* = 0.48) than those in the unhealthy condition (*M* = -1.71, *SD* = 0.54), *t*(190) = 52.11, *p* ≤ 0.001, *d* = 7.53.

#### Environmental Attitude Moderates the Effect of Past Environmental Actions on Some Intentions

Multiple regression analyses examined the effects of the recall manipulation (environmentally friendly vs. unfriendly behavior), environmental attitude, and their interaction on pro-environmental intentions and interest in apps. We tested two models for each dependent variable. In the first step, environmental attitude and the recalled behavior were entered as predictors. In the second step, the interaction term (Recall × Attitude) was added to the model. If adding the interaction term resulted in a statistically significant improvement to the model, we used the Johnson-Neyman conditional analysis (Spiller et al., 2013), made available through the R package jtools (Long, 2018), to identify the range of the environmental attitude for which the simple effect of the recall manipulation was significant. Simple slope analyses were then used to better understand the interactions (Cohen et al., 2003; Spiller et al., 2013).

##### Interaction effects

To test the prediction that attitude strength would influence the extent of positive and negative spillover, we first explored potential interaction effects. For two (of five) intentions, the effect of the recall manipulation depended on the strength of participants’ environmental attitude (Table 2).

**Table 2:** Direct and interactive effects of environmental attitude and recalled behavior on intentions and interest in apps, Study 1.

	Step 1			Step 2			
	*B*	*95% CI*	*R*^2^	*B*	*95% CI*	*R*^2^	*ΔR*^2^
**I1: Composting**							
Attitude	0.91***	[0.56, 1.26]	0.15	0.47^$^	[–0.02, 0.96]	0.18	0.03*
Recall manipulation	–0.64^$^	[–1.28, 0.01]		–0.77*	[–1.42, –0.13]		
Recall × attitude				0.87*	[0.18, 1.56]		
**I2: Cleaning agents**							
Attitude	0.99***	[0.74, 1.24]	0.27	0.96***	[0.60, 1.32]	0.27	0.00
Recall manipulation	0.09	[–0.37, 0.56]		0.08	[–0.39, 0.56]		
Recall × attitude				0.07	[–0.44, 0.57]		
**I3: Switching off electronic devices**							
Attitude	0.96***	[0.71, 1.20]	0.26	0.78***	[0.43, 1.14]	0.27	0.01
Recall manipulation	–0.43^$^	[–0.88, 0.03]		–0.48*	[–0.94, –0.02]		
Recall × attitude				0.33	[–0.16, 0.82]		
**I4: Local food**							
Attitude	0.54***	[0.38, 0.71]	0.19	0.44***	[0.19, 0.68]	0.19	0.01
Recall manipulation	–0.04	[–0.36, 0.27]		–0.07	[–0.39, 0.25]		
Recall × attitude				0.20	[–0.14, 0.54]		
**I5: Switching off lights**							
Attitude	0.25***	[0.13, 0.38]	.09	0.09	[–0.09, 0.26]	0.12	0.03*
Recall manipulation	–0.05	[–0.28, 0.18]		–0.10	[–0.33, 0.13]		
Recall × attitude				0.32*	[0.07, 0.56]		
**A1: Saving energy at work**							
Attitude	0.51***	[0.25, 0.77]	0.10	0.53**	[0.15, 0.90]	0.10	0.00
Recall manipulation	–0.44^$^	[–0.93, 0.04]		–0.44^$^	[–0.94, 0.05]		
Recall × attitude				–0.02	[–0.55, 0.50]		
**A2: Saving energy at home**							
Attitude	0.34**	[0.12, 0.56]	0.06	0.26	[–0.06, 0.58]	0.06	0.00
Recall manipulation	–0.24	[–0.65, 0.17]		–0.27	[–0.69, 0.15]		
Recall × attitude				0.16	[–0.29, 0.60]		
**A3: Reduce CO_2_**							
Attitude	0.61***	[0.37, 0.86]	0.13	0.56**	[0.21, 0.92]	0.13	0.00
Recall manipulation	–0.35	[–0.80, 0.11]		–0.36	[–0.83, 0.10]		
Recall × attitude				0.09	[–0.40, 0.59]		


*Environmentally unfriendly behavior = 0, environmentally friendly behavior = 1. ^∗∗∗^*p* < 0.001, ^∗∗^*p* < 0.01, ^∗^*p* < 0.05, ^$^*p* < 0.10.*

**FIGURE 2:**
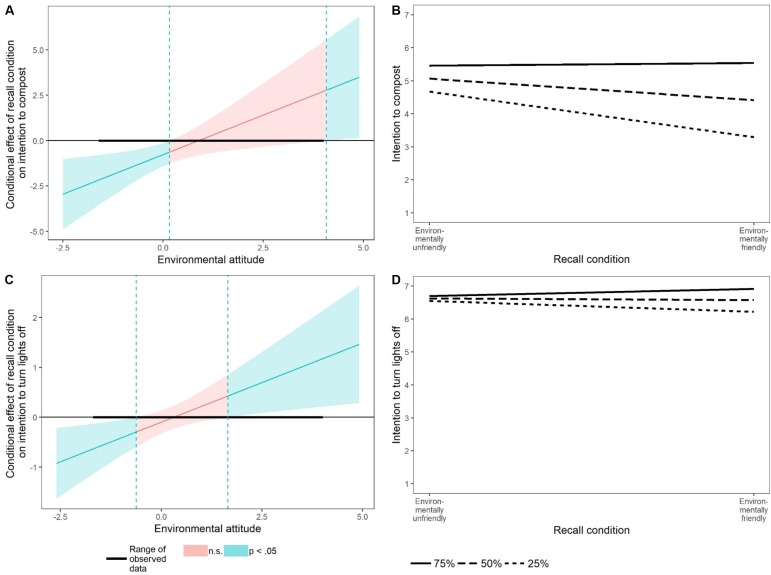
Panels **(A,C)** show the levels of environmental attitude for which recalling an environmentally friendly versus unfriendly behavior had a statistically significant effect on intention (Johnson-Neyman technique). Panels **(B,D)** show simple slopes of the effect of recalling an environmentally friendly versus unfriendly behavior on intentions for the median of the lower, middle, and upper terciles of environmental attitude.

The first interaction was found when the intention to compost green waste was used as the dependent variable (Table 2). Analysis of this interaction with the Johnson-Neyman technique showed that the recall manipulation had an effect only on participants with attitude scores less than 0.16 (i.e., the 53rd percentile; Figure 2A).^2^ The simple slopes for participants with strong attitudes (75th percentile) showed that these participants were equally motivated to compost regardless of whether they had recalled an environmentally friendly versus unfriendly action (*B* = 0.08, *SE* = 0.43, *p* = 0.85; Figure 2B). By contrast, those with medium or weak attitudes less strongly intended to compost when they had recalled an environmentally friendly compared to an environmentally unfriendly action (50th percentile: *B* = -0.65, *SE* = 0.32, *p* = 0.04; 25th percentile: *B* = -1.38, *SE* = 0.44, *p* < 0.001; Figure 2B).

The second interaction effect was found when participants indicated whether they intended to turn off the lights when leaving a room (Table 2). Using the Johnson-Neyman technique, it was found that recalling either an environmentally friendly or an unfriendly behavior significantly predicted the intention to turn off lights for participants who scored lower than -0.62 or higher than 1.66 on environmental attitude (Figure 2C). More specifically, the simple slopes again show that participants with a weak attitude (25th percentile) less strongly intended to turn off the lights after recalling an environmentally friendly than an environmentally unfriendly behavior (*B* = -0.33, *SE* = 0.16, *p* = 0.04; Figure 2D). By contrast, recalling either an environmentally friendly or unfriendly behavior did not have any effect on participants with medium or strong environmental attitudes, respectively (50th percentile: *B* = -0.05, *SE* = 0.11, *p* = 0.65; 75th percentile: *B* = 0.22, *SE* = 0.15, *p* = 0.16; Figure 2D). However, for 16 participants with an extremely environmentally friendly attitude (>1.66, 95th percentile), recalling an environmentally friendly behavior increased the intention to turn off lights compared to those who recalled a negative behavior (*B* = 0.52, *SE* = 0.25, *p* = 0.04).

We also tested for possible interactions between the recall manipulation and environmental attitude on participants’ interest in using three pro-environmental apps. None were statistically significant.

##### Direct effects of the recall manipulation and environmental attitude

Because the absence of statistically significant interaction effects implies that direct effects can be meaningfully interpreted, we examined whether the recall manipulation and environmental attitude had a direct influence on the dependent variables where the two predictors did not interact. Of eight dependent variables, there were no direct effects of the recall manipulation significant at the 5% level. However, it was found that the stronger participants’ level of environmental attitude, the more they were motivated to protect the environment and the more they were interested in relevant apps. This direct effect was found for all eight dependent variables.

Taken together, these results provide some support for our hypothesis. The patterns of the interactions are consistent with the prediction that participants with a weak environmental attitude would be affected by the valence of the recalled behavior such that they would be less motivated to engage in environmentally friendly behavior after recalling an environmentally friendly behavior (negative spillover). Among those with an *extremely* positive environmental attitude, the stronger intention to turn lights off after recalling an environmentally friendly action is an example of positive spillover.

#### Health Attitude Does Not Moderate the Effect of Past Health Behavior

##### Interaction effects

Following the same analytic approach, the prediction that a strong health attitude would increase the likelihood of positive spillover and reduce the likelihood of negative spillover was not confirmed. Health attitude did not moderate the effect of recalling an healthy or unhealthy behavior with respect to any of the five health intentions or interest in health-related apps (Table 3).

**Table 3:** Direct and interactive effects of health attitude and recalled behavior on intentions and interest in apps, Study 1.

	Step 1			Step 2			
	*B*	95% CI	*R*^2^	*B*	95% CI	*R*^2^	*ΔR*^2^
**I1: Treating myself with a snack**							
Attitude	0.35^$^	[–0.03, 0.74]	0.02	0.11	[–0.46, 0.68]	0.03	0.01
Recall manipulation	0.12	[–0.35, 0.59]		0.06	[–0.42, 0.54]		
Recall × attitude				0.45	[–0.32, 1.22]		
**I2: Taking time to relax**							
Attitude	0.42*	[0.09, 0.74]	0.03	0.49^$^	[–0.00, 0.98]	0.03	0.00
Recall manipulation	–0.04	[–0.43, 0.35]		–0.02	[–0.42, 0.38]		
Recall × attitude				–0.13	[–0.78, 0.53]		
**I3: Exercising at least 2 h/week**							
Attitude	0.95***	[0.58, 1.31]	0.12	0.76**	[0.22, 1.30]	0.13	0.00
Recall manipulation	–0.04	[–0.48, 0.40]		–0.08	[–0.53, 0.37]		
Recall × attitude				0.35	[–0.38, 1.08]		
**I4: Drinking less than 1 glass/day**							
Attitude	0.26	[–0.29, 0.81]	0.01	0.31	[–0.48, 1.10]	0.01	0.00
Recall manipulation	0.10	[–0.57, 0.76]		0.11	[–0.57, 0.78]		
Recall × attitude				–0.09	[–1.20, 1.02]		
**I5: Prepare at least 1 fresh meal/day**							
Attitude	0.90***	[0.55, 1.26]	0.12	0.96***	[0.43, 1.49]	0.12	0.00
Recall manipulation	0.31	[–0.13, 0.74]		0.32	[–0.13, 0.76]		
Recall × attitude				–0.10	[–0.83, 0.62]		
**A1: How to keep a healthy diet**							
Attitude	0.69***	[0.39, 1.00]	0.12	0.63**	[0.19, 1.08]	0.12	0.00
Recall manipulation	–0.36^$^	[–0.73, 0.01]		–0.37^$^	[–0.75, 0.00]		
Recall × attitude				0.11	[–0.50, 0.72]		
**A2: Relaxation techniques**							
Attitude	0.29	[–0.07, 0.66]	0.02	0.35	[–0.19, 0.90]	0.02	0.00
Recall manipulation	0.15	[–0.30, 0.60]		0.16	[–0.30, 0.62]		
Recall × attitude				–0.11	[–0.85, 0.63]		
**A3: More physical activity**							
Attitude	0.50*	[0.09, 0.90]	0.04	0.34	[–0.26, 0.94]	0.04	0.00
Recall manipulation	–0.31	[–0.80, 0.18]		–0.35	[–0.85, 0.15]		
Recall × attitude				0.30	[–0.51, 1.11]		


*Unhealthy behavior = 0, healthy behavior = 1. ^∗∗∗^*p* < 0.001, ^∗∗^*p* < 0.01, ^∗^*p* < 0.05, ^$^*p* < 0.10.*

##### Direct effects of the recall manipulation and health attitude

The recall manipulation again did not affect any of the dependent variables at the 5% significance level. Health attitude was, however, positively related to three behavioral intentions and interest in two apps.

### Discussion

Study 1 tested the hypothesis that attitude strength would moderate the effect of an initial behavior on subsequent behaviors. We expected that those with a strong (favorable) attitude would be equally motivated to engage in additional goal-conducive behaviors after recalling either a goal-consistent (environmentally friendly/healthy) or a goal-inconsistent past behavior (environmentally unfriendly/unhealthy), whereas those with a weak attitude would be less motivated to engage in further behaviors after recalling a goal-consistent compared to a goal-inconsistent behavior.

The results of Study 1 provided initial support for this prediction in two of five pro-environmental intentions but in none of the health-related intentions. One possible explanation for why the predicted interaction was not found in more dependent variables is that Study 1 did not have sufficient statistical power to detect the interaction effect. To obtain a rough estimate of the power of Study 1, we conducted a power analysis using the special *F*-test assessing the increase in explained variance due to the interaction with three predictors (i.e., attitude, dummy representing the experimental condition, and their interaction) and a significance level of 0.05 (Faul et al., 2009). Based on these assumptions, the sample size of the two regression analyses (*N*s = 185, 193) provided high power (1 - β > 0.98) for finding a conventional medium-sized effect (i.e., |*B*| = 0.30) but only weak power (1 - β = 0.27/0.28) for finding a small effect (i.e., |*B*| = 0.10). The power analysis suggests that a larger sample size is necessary to find small interaction effects.

Another limitation of Study 1 was that the control condition was recalling a goal-inconsistent (unhealthy or environmentally unfriendly) behavior rather than a more neutral task. A weakness of this design is that it is impossible to conclude whether effects of the experimental conditions originate uniquely from recalling a goal-consistent behavior, a goal-inconsistent behavior, or from their combined effects (Mullen and Monin, 2016). To illustrate, the finding that 16 participants with an extremely strong pro-environmental attitude were more motivated to turn lights off after recalling a goal-consistent action (environmentally friendly) could stem from an increase in this intention among those who recalled a goal-consistent behavior, from a decrease among those who recalled a goal-inconsistent behavior – or both. Although all three explanations are logically possible, from a theoretical perspective it seems somewhat implausible that those with the most extreme pro-environmental attitude would act against their goal after an environmentally friendly action. Ultimately, however, this is an empirical question that requires empirical testing and can best be investigated with an additional neutral condition.

A further limitation of Study 1 is that the sample consisted mainly of female students. Consequently, environmental and health attitudes may have been more homogeneous than in the general adult population. Without a more representative sample, the findings of Study 1 might be limited to well-educated female students.

## Study 2

Study 2 aimed to replicate the findings of Study 1 and address its shortcomings by adding a neutral control condition and by using a larger and demographically more heterogeneous sample. We used the neutral control condition as a baseline and examined the moderating effect of attitude strength on recalling a goal-inconsistent (environmentally unfriendly/unhealthy) or goal-consistent (environmentally friendly/healthy) behavior.

We expected that participants with a strong attitude would be more motivated to engage in goal-conducive behaviors after recalling either a goal-consistent or goal-inconsistent behavior than after recalling a neutral behavior. The prediction is based on the following assumptions: when such participants carry out a behavior that is relevant to their attitude, it increases (a) the salience of the attitude and (b) the relationships between different attitude-relevant behaviors and how they are relevant to the underlying attitude; and (c) if such participants carry out a behavior inconsistent with their attitude, they experience cognitive dissonance. Regarding participants with weak attitudes, we predicted that they would feel that they had “done enough” and be less motivated to engage in further similar behaviors after recalling a goal-consistent behavior compared to a neutral behavior. For these participants, previous environmentally unfriendly or unhealthy actions are unlikely to lead to cognitive dissonance because they do not conflict with attitudes. We therefore did not expect motivation to differ after recalling a goal-inconsistent behavior relative to recalling a neutral behavior.

### Materials and Methods

#### Procedure

The general procedure was the same as Study 1. Data were again collected through Qualtrics at two points in time in 2018. At time 1, participants answered questions regarding their environmental and health attitudes and socio-demographic questions.

At time 2 (8–12 days later), participants completed one of five recall conditions, to which they were assigned randomly. After answering two sets of questions that are beyond the scope of Study 2 (i.e., relating to possible additional moral processes), participants answered the questions used as dependent variables. Finally, they were thanked and debriefed.

#### Participants

A power analysis using the special *F*-test assessing the increase in explained variance due to the interaction with five predictors (i.e., attitude, two dummies representing the experimental conditions, and their interactions; Faul et al., 2009) suggested that to find a small-to-medium effect (|*B*| = 0.15) with 90% power at the 5% level, at least 553 participants are required for an experimental design with three groups. To be able to conduct the analysis in two contexts (environment and health), we increased the target sample size proportionally and aimed for a total sample of *N* = 922.

The United States-based sample was recruited via Amazon Turk. Those who participated in both parts of the survey were paid US $4. In total, 1,208 participants started the survey at time 1. Of those, 26 were removed due to a missing personal identifier. Eighteen were removed because they participated more than once (in which case we discarded the participation that included more missing values, and in case of a similar amount of missing values, the second participation). A further 38 participants were removed because they responded to fewer than 20% of the questions.

Of all participants who finished the survey at time 1, 1,003 accessed the study at time 2. Ten participants participated twice; we again excluded the answers from the participation that included more missing values, and in case of a similar amount of missing values, the second participation. A further 37 participants were removed because they responded to less than 20% of the questions.

Some 174 participants were excluded as they did not take part in both parts of the study. To ensure good data quality, we again retained only participants (a) who passed the seriousness check (Reips, 2002), (b) whose participation time in both surveys lasted at least one third of the sample’s median time (10.55 min at time 1, 10.19 min at time 2), (c) who provided a semantically meaningful answer in the recall task (judged by three independent raters), and (d) who passed the attention checks that were included in both parts of the study. Based on these criteria, 25 participants were excluded. The mean age of participants who met the criteria (*N* = 929) was 37.42 (*SD* = 12.01). The proportion of women was approximately 65%. Of participants who revealed their academic background, for 10.1% the highest degree was high school or lower, 20.1% partially completed college, 13.5% fully completed college, 39.6% had a bachelor’s degree, and 16.7% a master’s or Ph.D. degree.

A comparison of the 199 participants who either did not participate in the survey both times (*N* = 174) or who did participate but were excluded to ensure good data quality and the 929 participants who were retained for the analyses did not reveal any differences in environmental or health attitudes (*t*-tests, *p*s = 0.17, 0.60). The proportion of participants who dropped out or were excluded was not associated with experimental condition, χ^2^(4) = 1.75, *p* = 0.782.

#### Manipulation

Participants were randomly assigned to one of five experimental conditions. In addition to the four conditions used in Study 1, a control condition was included in which participants were asked to recall their routine on a typical Tuesday (Jordan et al., 2011; Cornelissen et al., 2013). In all conditions, participants were instructed to take 5–10 min to write down their action or routine in detail (Jordan et al., 2011; Weibel et al., 2014).

To examine whether the manipulation had the intended effect, three coders blind to condition rated how environmentally friendly and healthy the recalled deeds were (seven-point scale: -3 = very environmentally harmful or unhealthy, +3 = very environmentally friendly or healthy). Interrater reliability was high (intraclass correlation coefficient [ICC]_environmentallyfriendly_] = 0.88, ICC_healthy_ = 0.89). The ratings of the coders were averaged into an environmental friendliness and a healthiness scale.

#### Moderators

*Environmental attitude* was measured with 47 items (see Supplementary Table 1) from Kaiser and Wilson (2004). Of the 47 items, 30 were presented in a five-point frequency format. The responses to these items were recoded into a dichotomous format by collapsing “never,” “seldom,” and “occasionally” into “unreliable pro-environmental engagement,” and “often” and “always” into “reliable pro-environmental engagement.” The remaining 17 items were presented in a yes/no format. Nineteen behaviors represented environmentally unfriendly activities and were recoded prior to analysis. The dichotomization, calibration of the behavior scale, and the estimation of person scores were based on the classical Rasch model and in line with previous calibrations of the same instrument (Kaiser and Wilson, 2004). All behavior items were found to fit the model very well (infit mean square values < 1.29; for reference values, see Bond and Fox, 2007). The Rasch-model-based reliability estimate of the measure was *rel* = 0.74.

*Health attitude* was measured with 44 items from Byrka and Kaiser (2013) and nine newly developed items (Supplementary Table 2). For 27 items, a five-point frequency scale was used; then responses were dichotomized as for the environmental scale. The remaining 24 items were in a yes/no format. Nine items represented unhealthy behaviors and were recoded prior to analysis. All behavior items fit the model very well (infit mean square values < 1.23). The Rasch-model-based reliability estimate was *rel* = 0.77.

#### Dependent Variables

To assess the extent of positive and negative spillover, we used four types of dependent variables. First, participants indicated on a seven-point scale (1 = very unlikely, 7 = very likely) how likely they are to engage in 17 behaviors in the near future. Of these behavioral intentions, eight were related to the environment and nine to their personal health (Table 4).

**Table 4:** Descriptive statistics for behavioral intentions (I1–I8), petitions (P1–P6), interest in behavior tips, and donations in the contexts of environment and health, Study 2.

	*Mean*	*Median*	*SD*	*Range*
**Environmental protection**				
I1: Switching off electronic devices instead of leaving them on stand-by	4.02	4	1.87	1–7
I2: Forego air travel and instead choose a means of transport with less negative effects on the environment	3.85	4	1.92	1–7
I3: Buy ecologically produced food	3.92	4	1.55	1–7
I4: Only eat seasonal produce	3.83	4	1.68	1–7
I5: Boycott products from businesses that harm the environment	3.71	4	1.7	1–7
I6: Buy the environmentally friendly alternative of a product	4.52	5	1.53	1–7
I7: Always recycle plastic bottles (even in public places)	5.35	6	1.61	1–7
I8: Join an environmental group	2.7	2	1.59	1–7
P1: Fee for paper cups	3.41	3	1.96	1–7
P2: Plastic bag tax	4.17	5	2.17	1–7
P3: Ban non-sustainable palm oil	4.32	5	1.97	1–7
P4: Ban plastic dishes	3.87	4	2.07	1–7
P5: Invest in renewable energy	5.2	6	1.92	1–7
P6: No drilling in arctic national wildlife refuge	5.01	6	2.03	1–7
S1: Interest in information sheet	0.6	1	0.49	0–1
D1: Amount environmental donation	0.15	0	0.47	0–4
**Health**				
I1: Eat four to five servings of fruit/vegetables per day	4.62	5	1.67	1–7
I2: Avoid snacks high in calories (e.g., chips, chocolate)	4.15	4	1.79	1–7
I3: Choose lean over fatty food options	4.81	5	1.58	1–7
I4: Regularly take the stairs instead of the elevator	4.89	5	1.64	1–7
I5: Do 150 min/week of moderate physical activity (gentle swimming, golf, horseback riding)	4.46	5	1.89	1–7
I6: Do 75 min/week of vigorous physical activity (joggin, cycling, aerobics, competitive tennis)	4.33	5	1.91	1–7
I7: Have regular health check-ups (dental hygiene, gynecologist, cancer checks)	4.96	5	1.68	1–7
I8: Drink no more than two beers or similar per week	5.37	7	2.11	1–7
I9: Use sunscreen consistently when exposed to the sun	4.73	5	1.86	1–7
S1: Interest in information sheet	0.61	1	0.49	0–1


Second, participants indicated on a seven-point scale (1 = very unlikely, 7 = very likely) how likely they were to sign nine petitions from online sites ^3^^,^^4^ . Of the nine petitions, six were related to environmental protection (Table 4) and three to improving health.

Third, participants indicated (yes/no) whether they were interested in receiving tips about pro-environmental or healthy behaviors. Fourth, they were given the chance to donate any part of their reimbursement to either an organization for the protection of the environment (Table 4) or the promotion of health.

We did not examine any effects on support for health-related petitions or donations. This is because health attitude focuses on people’s *personal* health. This makes it difficult or impossible to anticipate any systematic relationship between health attitude and decisions that focus predominantly on promoting *others’* health.

### Results

#### Levels of Environmental and Health Attitudes in the Five Experimental Conditions

The random allocation of participants to the five conditions was successful with respect to the strength of the attitudes: The levels of environmental [*F*(4,924) = 1.39, *p* = 0.235, η^2^ = 0.01] and health attitude [*F*(4,924) = 1.59, *p* = 0.175, η^2^ = 0.01] were not statistically different in the five conditions.

#### Manipulation Checks

##### Environmental behavior

The manipulation check showed that the recall manipulation had the intended effect. Coders rated the recalled environmental behaviors in the three conditions differently [*F*(2,535) = 1814.00, *p* < 0.001, η^2^ = 0.87]. *Post hoc* comparisons using the Tukey HSD test indicated that coders rated the recalled action as more positive in the environmentally friendly condition (*M* = 1.50, *SD* = 0.56) than in the control condition (*M* = 0.00, *SD* = 0.00) and the environmentally unfriendly condition (*M* = -1.21, *SD* = 0.48), and as more positive in the control condition than in the environmentally unfriendly condition.

##### Health behavior

The recall manipulation also had the intended effect with respect to health. Coders rated the recalled behaviors in the three conditions differently [*F*(2,532) = 2442.00, *p* < 0.001, η^2^ = 0.90]. *Post hoc* comparisons using the Tukey HSD test indicated that coders rated the recalled health action as more positive in the healthy condition (*M* = 1.43, *SD* = 0.48) than in the control condition (*M* = 0.00, *SD* = 0.00) and the unhealthy condition (*M* = -1.29, *SD* = 0.42), and as more positive in the control than the unhealthy condition.

#### Environmental Attitude Moderates the Effect of Past Environmental Actions on One Petition and Has a Direct Positive Effect on All Dependent Variables

To examine the effects of the recall manipulation, environmental attitude, and their interaction on intentions and support for petitions, we used the same multiple linear regression approach as in Study 1. Because of the dichotomous answer format of the pro-environmental information sheet, we used a logistic regression analysis to examine effects on this dependent variable. Furthermore, only 14% of the sample donated to any organization, resulting in a high frequency of zero data points and a strongly positively skewed distribution. We therefore used negative binomial regression analyses when donations to a pro-environmental organization was the dependent variable (Carrico et al., 2018).

##### Interaction effects

**FIGURE 3:**
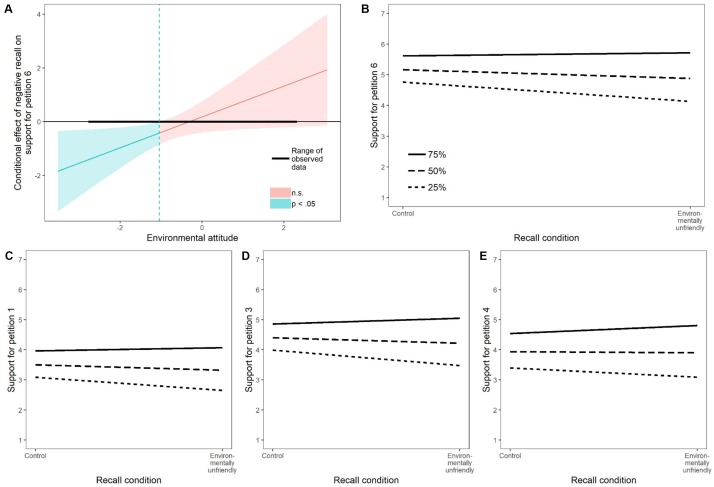
Panel **(A)** shows the level of environmental attitude for which recalling an environmentally unfriendly behavior versus a control condition had a statistically significant effect on petition 6 (Johnson-Neyman technique). Panel **(B)** shows simple slopes of the effect of recalling an environmentally unfriendly behavior versus a control condition on support for petition 6 for the median of the lower, middle, and upper terciles of environmental attitude. Panels **(C–E)** show the same trend (significant at the 10% significance level) as panel **(B)** for three additional petitions.

For one (of six) petitions, the effect of the environmentally unfriendly recall manipulation depended on the strength of participants’ environmental attitude: The significant interaction was found when petition 6 (no drilling in the arctic national wildlife refuge) was used as the dependent variable and the terms that represented the interaction between environmental attitude and participants who either recalled a typical Tuesday (control group) or an environmentally unfriendly behavior were included as predictors (Table 5). Analysis of this interaction with the Johnson-Neyman technique showed that the environmentally unfriendly recall manipulation had an effect only on participants with attitude scores less than -1.04 (39th percentile), not for participants whose environmental attitude was equal to or greater than -1.04 (Figure 3A). The simple slopes for participants with a weak environmental attitude (25th percentile) showed that they less strongly intended to sign the petition when they had recalled an environmentally unfriendly compared to a neutral behavior (*B* = -0.63, *SE* = 0.26, *p* = 0.02; Figure 3B). By contrast, those with a strong or medium attitude were equally motivated to sign the petition after recalling a neutral or an environmentally unfriendly deed (75th percentile: *B* = 0.10, *SE* = 0.27, *p* = 0.71; 50th percentile: *B* = -0.28, *SE* = 0.20, *p* = 0.17; Figure 3B).

**Table 5:** Direct and interactive effects of environmental attitude and recalled behavior on intentions, willingness to sign petitions, interest in information sheet and amount donated, Study 2.

	Step 1			Step 2			
	*B*	*95% CI*	*R*^2^	*B*	*95% CI*	*R*^2^	*ΔR*^2^
**I1: Switch off electronic devices**							
Attitude	0.90***	[0.71, 1.09]	0.16	1.04***	[0.72, 1.36]	0.16	0.00
Recall environmentally Friendly	0.69***	[0.34, 1.03]		0.58*	[0.08, 1.07]		
Recall environmentally Unfriendly	0.11	[–0.24, 0.46]		–0.13	[–0.64, 0.38]		
Recall environmentally Friendly × attitude				–0.15	[–0.61, 0.30]		
Recall environmentally Unfriendly × attitude				–0.30	[–0.76, 0.17]		
**I2: Switch from air travel other means of transport**							
Attitude	0.63***	[0.43, 0.84]	0.06	0.77***	[0.42, 1.12]	0.07	0.01
Recall environmentally Friendly	0.23	[–0.16, 0.61]		–0.11	[–0.66, 0.43]		
Recall environmentally Unfriendly	0.07	[–0.32, 0.45]		0.11	[–0.46, 0.67]		
Recall environmentally Friendly × attitude				–0.44^$^	[–0.94, 0.07]		
Recall environmentally Unfriendly × attitude				0.02	[–0.49, 0.53]		
**I3: Buy ecologically produced food**							
Attitude	0.92***	[0.77, 1.08]	0.21	0.86***	[0.60, 1.11]	0.21	0.01
Recall environmentally Friendly	0.24^$^	[–0.04, 0.52]		0.18	[–0.22, 0.58]		
Recall environmentally Unfriendly	–0.02	[–0.30, 0.26]		0.22	[–0.19, 0.63]		
Recall environmentally Friendly × attitude				–0.07	[–0.44, 0.30]		
Recall environmentally Unfriendly × attitude				0.29	[–0.09, 0.66]		
**I4: Eat seasonal produce**							
Attitude	0.66***	[0.49, 0.84]	0.09	0.57***	[0.28, 0.87]	0.09	0.00
Recall environmentally Friendly	0.09	[–0.23, 0.42]		0.15	[–0.31, 0.61]		
Recall environmentally Unfriendly	0.03	[–0.30, 0.36]		0.19	[–0.28, 0.67]		
Recall environmentally Friendly × attitude				0.08	[–0.35, 0.51]		
Recall environmentally Unfriendly × attitude				0.21	[–0.23, 0.64]		
**I5: Boycott products**							
Attitude	1.09***	[0.92, 1.25]	0.24	1.00***	[0.73, 1.28]	0.24	0.00
Recall environmentally Friendly	0.22	[–0.08, 0.52]		0.20	[–0.22, 0.62]		
Recall environmentally Unfriendly	0.13	[–0.18, 0.43]		0.36	[–0.08, 0.80]		
Recall environmentally Friendly × attitude				–0.02	[–0.41, 0.38]		
Recall environmentally Unfriendly × attitude				0.29	[–0.11, 0.69]		
**I6: Buy the environmentally friendly alternative of a product**							
Attitude	0.88***	[0.73, 1.03]	0.20	0.96***	[0.70, 1.21]	0.21	0.01*
Recall environmentally Friendly	0.37**	[0.09, 0.65]		0.10	[–0.30, 0.49]		
Recall environmentally Unfriendly	–0.04	[–0.32, 0.24]		0.08	[–0.32, 0.49]		
Recall environmentally Friendly × attitude				–0.35^$^	[–0.72, 0.01]		
Recall environmentally Unfriendly × attitude				0.13	[–0.24, 0.50]		
**I7: Always recycle plastic bottles**							
Attitude	0.76***	[0.60, 0.93]	0.19	0.89***	[0.61, 1.16]	0.19	0.00
Recall environmentally Friendly	0.47**	[0.18, 0.77]		0.23	[–0.19, 0.65]		
Recall environmentally Unfriendly	–0.46**	[–0.76, -0.16]		–0.48*	[–0.91, -0.04]		
Recall environmentally Friendly × attitude				–0.32	[–0.71, 0.07]		
Recall environmentally Unfriendly × attitude				–0.04	[–0.44, 0.35]		
**I8: Join an environmental group**							
Attitude	0.90***	[0.74, 1.06]	0.18	1.01***	[0.73, 1.28]	0.18	0.00
Recall environmentally Friendly	–0.09	[–0.38, 0.21]		–0.29	[–0.71, 0.13]		
Recall environmentally Unfriendly	–0.10	[–0.41, 0.20]		–0.13	[–0.57, 0.30]		
Recall environmentally Friendly × attitude				–0.26	[–0.66, 0.13]		
Recall environmentally Unfriendly × attitude				–0.06	[–0.45, 0.34]		
**P1: Fee for paper cups**							
Attitude	0.88***	[0.67, 1.08]	0.12	0.69***	[0.35, 1.04]	0.12	0.00
Recall environmentally Friendly	0.12	[–0.26, 0.49]		0.22	[–0.31, 0.76]		
Recall environmentally Unfriendly	–0.18	[–0.56, 0.20]		0.16	[–0.39, 0.72]		
Recall environmentally Friendly × attitude				0.15	[–0.34, 0.65]		
Recall environmentally Unfriendly × attitude				0.43^$^	[–0.07, 0.93]		
**P2: Plastic bag tax**							
Attitude	1.00***	[0.78, 1.23]	0.12	0.87***	[0.49, 1.24]	0.13	0.00
Recall environmentally Friendly	0.05	[–0.36, 0.46]		0.06	[–0.52, 0.64]		
Recall environmentally Unfriendly	–0.02	[–0.44, 0.40]		0.31	[–0.29, 0.92]		
Recall environmentally Friendly × attitude				0.03	[–0.51, 0.57]		
Recall environmentally Unfriendly × attitude				0.41	[–0.14, 0.96]		
**P3: Ban non-sustainable palm oil**							
Attitude	0.86***	[0.65, 1.07]	0.11	0.69***	[0.33, 1.04]	0.12	0.01^$^
Recall environmentally Friendly	0.15	[–0.23, 0.54]		0.13	[–0.42, 0.67]		
Recall environmentally Unfriendly	–0.19	[–0.58, 0.20]		0.26	[–0.30, 0.83]		
Recall environmentally Friendly × attitude				–0.01	[–0.52, 0.49]		
Recall environmentally Unfriendly × attitude				0.56*	[0.04, 1.07]		
**P4: Ban plastic dishes**							
Attitude	1.06***	[0.85, 1.27]	0.15	0.90***	[0.55, 1.26]	0.16	0.01
Recall environmentally Friendly	0.04	[–0.35, 0.42]		0.07	[–0.48, 0.61]		
Recall environmentally Unfriendly	–0.04	[–0.44, 0.35]		0.33	[–0.24, 0.89]		
Recall environmentally Friendly × attitude				0.06	[–0.45, 0.56]		
Recall environmentally Unfriendly × attitude				0.45^$^	[–0.06, 0.97]		
**P5: Invest in renewable energy**							
Attitude	0.63***	[0.43, 0.84]	0.07	0.55**	[0.21, 0.88]	0.07	0.00
Recall environmentally Friendly	–0.03	[–0.40, 0.34]		–0.06	[–0.58, 0.47]		
Recall environmentally Unfriendly	–0.23	[–0.60, 0.15]		0.02	[–0.52, 0.57]		
Recall environmentally Friendly × attitude				–0.02	[–0.51, 0.47]		
Recall environmentally Unfriendly × attitude				0.31	[–0.19, 0.80]		
**P6: No drilling in arctic national wildlife refuge**							
Attitude	0.79***	[0.58, 1.01]	0.10	0.68***	[0.32, 1.03]	0.11	0.01*
Recall environmentally Friendly	0.14	[–0.25, 0.53]		–0.02	[–0.57, 0.53]		
Recall environmentally Unfriendly	–0.30	[–0.70, 0.09]		0.17	[–0.40, 0.74]		
Recall environmentally Friendly × attitude				–0.18	[–0.69, 0.33]		
Recall environmentally Unfriendly × attitude				0.57*	[0.06, 1.09]		
**S1: Information sheet y/n^a^**							
Attitude	0.48***	[0.24, 0.73]	0.03	0.62**	[0.19, 1.09]	0.03	0.00
Recall environmentally Friendly	–0.22	[–0.65, 0.22]		–0.45	[–1.14, 0.21]		
Recall environmentally Unfriendly	–0.44*	[–0.87, -0.01]		–0.53	[–1.23, 0.16]		
Recall environmentally Friendly × attitude				–0.28	[–0.90, 0.32]		
Recall environmentally Unfriendly × attitude				–0.11	[–0.74, 0.50]		
**D1: Amount environmental donation^b^**							
Attitude	0.84***	[ 0.57, 1.12]	0.07	0.63**	[0.17, 1.08]	0.07	0.00
Recall environmentally Friendly	0.15	[–0.41, 0.71]		0.28	[–0.30, 0.87]		
Recall environmentally Unfriendly	0.36	[–0.19, 0.92]		0.42	[–0.19, 1.02]		
Recall environmentally Friendly × attitude				0.44	[–0.21, 1.11]		
Recall environmentally Unfriendly × attitude				0.20	[–0.46, 0.87]		


*Environmentally unfriendly behavior = 0, environmentally friendly behavior = 1. ^∗∗∗^*p* < 0.001, ^∗∗^*p* < 0.01, ^∗^*p* < 0.05, *p* < 0.10. ^*a*^Logistic regression. ^*b*^Negative binomial regression.*

Similar trends were observed for petition 1 (fee for paper cups), petition 3 (ban unsustainable palm oil) and petition 4 (ban plastic dishes); however, with only marginally significant effects (Figure 3C–E). These patterns are not consistent with the prediction that after recalling an environmentally unfriendly versus a neutral behavior, participants with a strong attitude would increase their support for environmental policies, whereas participants with a weak attitude would be relatively unaffected by the two types of memories.

##### Direct effects of environmental attitude and the recall manipulation

When the valence of the recalled behavior was held constant, participants with a strong environmental attitude acted more environmentally friendly than participants with a weak environmental attitude. This direct effect was observed in all 16 dependent variables (Table 5) and is evident, for example, in the varying levels of support for petitions in Figure 3B–E.

Recalling a neutral versus an environmentally friendly or unfriendly behavior also had some direct effects on the environmental outcome variables: When controlling for the influence of environmental attitude, recalling an environmentally friendly (vs. neutral) behavior increased the motivation to engage in three pro-environmental behaviors (switch off electronic devices, buy eco-friendly products, and recycle plastic bottles). In other words, recalling an environmentally friendly deed promoted positive spillover across all levels of environmental attitude with respect to these intentions. When the intention to recycle plastic bottles was the dependent variable, this behavioral consistency was also observed in the other direction: Recalling an environmentally unfriendly (vs. neutral) behavior decreased the intention to recycle, irrespective of the strength of environmental attitude. Finally, behavioral consistency was found when participants who recalled an environmentally unfriendly behavior were asked if they wanted to receive tips about pro-environmental behavior: Compared to the neutral condition, they were less interested in receiving such information.

#### Health Attitude Has a Direct Positive Effect on All Dependent Variables

##### Interaction effects

**FIGURE 4:**
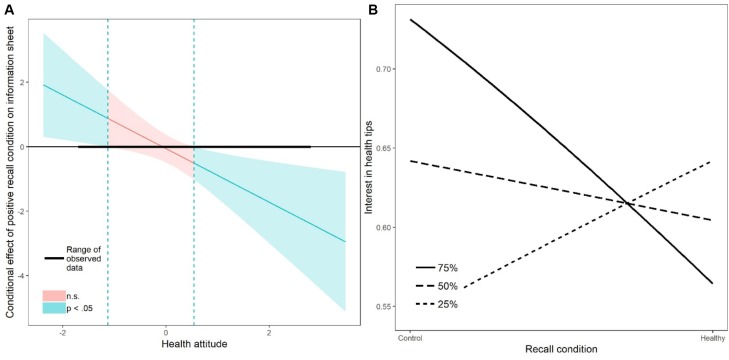
Panel **(A)** shows the level of health attitude for which recalling a healthy behavior versus a control condition had a statistically significant effect on the interest in an information sheet with health tips (Johnson-Neyman technique). Panel **(B)** shows simple slopes of the effect of recalling a healthy behavior versus a control condition on interest in an information sheet with health tips for the median of the lower, middle, and upper terciles of health attitude.

The prediction that a strong health attitude would increase the likelihood of positive spillover and reduce the likelihood of negative spillover after an initial healthy behavior was not confirmed (Table 6). There was even some evidence to suggest a detrimental influence of a strong health attitude. We found a significant interaction when interest in tips for how to live healthily was used as a dependent variable and the healthy (vs. neutral) recall manipulation, health attitude, and their interactions were used as predictors (Table 6). A decomposition of this interaction with the Johnson-Neyman technique showed that recalling a healthy behavior had an effect only on participants with attitude scores less than -1.13 (i.e., the 3rd percentile) and more than 0.55 (i.e., the 74th percentile; Figure 4A). The simple slopes for participants with strong attitudes (75th percentile) showed that these participants requested the information sheet less frequently when they had recalled a healthy compared to a neutral deed (*B* = -0.74, *SE* = 0.32, *p* = 0.02, Figure 4B). By contrast, those with moderate and weak health attitudes did not differ in their interest in the information when they had recalled a healthy or a neutral deed (50th percentile: *B* = -0.16, *SE* = 0.22, *p* = 0.46; 25th percentile: *B* = 0.41, *SE* = 0.30, *p* = 0.18; Figure 4B).

**Table 6:** Direct and interactive effects of health attitude and recalled behavior on intentions and interest in information sheet 2.

	Step 1			Step 2			
	*B*	*95% CI*	*R*^2^	*B*	*95% CI*	*R*^2^	*ΔR*^2^
**I1: Four to five servings of fruit/vegetables per day**							
Attitude	0.95***	[0.76, 1.13]	0.16	1.08***	[0.77, 1.39]	0.16	0.00
Recall healthy	–0.11	[–0.42, 0.20]		–0.07	[–0.39, 0.24]		
Recall unhealthy	–0.24	[–0.55, 0.07]		–0.21	[–0.53, 0.10]		
Recall healthy × attitude				–0.29	[–0.73, 0.16]		
Recall unhealthy × attitude				–0.13	[–0.57, 0.32]		
**I2: Avoid snacks high in calories**							
Attitude	0.89***	[0.69, 1.08]	0.13	0.81***	[0.49, 1.14]	0.14	0.01^$^
Recall healthy	0.41*	[0.07, 0.74]		0.42*	[0.08, 0.76]		
Recall unhealthy	0.22	[–0.11, 0.56]		0.17	[–0.17, 0.51]		
Recall healthy × attitude				–0.16	[–0.64, 0.32]		
Recall unhealthy × attitude				0.38	[–0.10, 0.86]		
**I3: Choose lean over fatty food options**							
Attitude	0.84***	[0.66, 1.01]	0.14	0.81***	[0.52, 1.10]	0.14	0.00
Recall healthy	–0.02	[–0.31, 0.28]		–0.01	[–0.31, 0.29]		
Recall unhealthy	–0.21	[–0.50, 0.08]		–0.24	[–0.53, 0.06]		
Recall healthy × attitude				–0.08	[–0.50, 0.34]		
Recall unhealthy × attitude				0.15	[–0.27, 0.57]		
**I4: Take the stairs instead of the elevator**							
Attitude	0.80***	[0.61, 0.98]	0.12	0.98***	[0.68, 1.28]	0.13	0.01
Recall healthy	0.08	[–0.22, 0.39]		0.13	[–0.17, 0.44]		
Recall unhealthy	–0.11	[–0.41, 0.20]		–0.08	[–0.39, 0.23]		
Recall healthy × attitude				–0.42^$^	[–0.86, 0.02]		
Recall unhealthy × attitude				–0.16	[–0.59, 0.28]		
**I5: Moderate physical activity**							
Attitude	1.08***	[0.87, 1.28]	0.16	0.96***	[0.62, 1.30]	0.16	0.00
Recall healthy	0.17	[–0.18, 0.52]		0.14	[–0.21, 0.49]		
Recall unhealthy	0.27	[–0.07, 0.62]		0.25	[–0.11, 0.61]		
Recall healthy × attitude				0.23	[–0.27, 0.74]		
Recall unhealthy × attitude				0.14	[–0.36, 0.64]		
**I6: Vigorous physical activity**							
Attitude	1.14***	[0.92, 1.35]	0.17	1.09***	[0.73, 1.44]	0.17	0.00
Recall healthy	–0.02	[–0.38, 0.33]		–0.02	[–0.38, 0.35]		
Recall unhealthy	–0.08	[–0.43, 0.28]		–0.11	[–0.48, 0.25]		
Recall healthy × attitude				–0.09	[–0.61, 0.43]		
Recall unhealthy × attitude				0.25	[–0.27, 0.76]		
**I7: Have regular health check-ups**							
Attitude	0.69***	[0.50, 0.88]	0.08	0.67***	[0.35, 0.99]	0.08	0.00
Recall healthy	0	[–0.32, 0.33]		0	[–0.33, 0.33]		
Recall unhealthy	0.1	[–0.22, 0.43]		0.1	[–0.23, 0.43]		
Recall healthy × attitude				0.02	[–0.45, 0.48]		
Recall unhealthy × attitude				0.05	[–0.41, 0.52]		
**I8: Drink maximum two drinks/week**							
Attitude	0.37**	[0.12, 0.62]	0.02	0.61**	[0.20, 1.03]	0.02	0.00
Recall healthy	0.01	[–0.41, 0.43]		0.05	[–0.38, 0.48]		
Recall unhealthy	–0.05	[–0.47, 0.37]		0.02	[–0.41, 0.45]		
Recall healthy × attitude				–0.27	[–0.88, 0.34]		
Recall unhealthy × attitude				–0.48	[–1.09, 0.12]		
**I9: Use sunscreen consistently**		
Attitude	0.73***	[0.52, 0.95]	0.08	0.70***	[0.34, 1.06]	0.08	0.00
Recall healthy	0.04	[–0.32, 0.41]		0.03	[–0.34, 0.40]		
Recall unhealthy	–0.25	[–0.62, 0.11]		–0.25	[–0.63, 0.12]		
Recall healthy × attitude				0.12	[–0.41, 0.65]		
Recall unhealthy × attitude				–0.01	[–0.53, 0.52]		
**S1: Information sheet^a^**							
Attitude	0.20	[–0.05, 0.46]	0.00	0.60**	[0.16, 1.06]	0.01	0.01*
Recall healthy	–0.14	[–0.56, 0.28]		–0.06	[–0.49, 0.37]		
Recall unhealthy	–0.13	[–0.55, 0.29]		–0.09	[–0.52, 0.34]		
Recall healthy × attitude				–0.83**	[–1,47, -2.11]		
Recall unhealthy × attitude				–0.36	[–0.99, 0.27]		


*Unhealthy behavior = 0, healthy behavior = 1. ^∗∗∗^*p* < 0.001, ^∗∗^*p* < 0.01, ^∗^*p* < 0.05, ^$^*p*< 0.10. ^*a*^Logistic regression.*

##### Direct effects of health attitude and the recall manipulation

Attitude was positively related to all nine health intentions; that is, the stronger a person’s health attitude, the more likely they were to act in a healthy way (Table 6). When controlling for the influence of attitude, recalling a healthy (vs. neutral) behavior increased the intention to avoid snacks high in calories (intention 2, Table 6). No other positive or negative spillover effects of the recall manipulation were found.

### Discussion

Study 2 provided little evidence for the expected moderating effect of attitude strength: In only two instances – when participants were asked whether they would support a petition against drilling in an arctic wildlife refuge and when they were asked whether they wanted to receive health tips – did the respective attitude moderate the effect of the recalled behavior at the 5% significance level.

What is more, these interactions were not entirely in line with our predictions: We expected that recalling a healthy (vs. a neutral) behavior would increase the interest in receiving health tips among those with a strong health attitude, but found that the recalled behavior decreased their interest in such tips. It is striking that the latter interaction was the only one across both studies in which those with a strong attitude *reduced* their efforts to act in line with their attitude.

To explain this unexpected pattern, we look to the content of the dependent variable: the choice to receive information. It could be argued that participants who have a strong health attitude tend to already know a lot about health. This expertise may have become particularly obvious after recalling a healthy behavior, which might in turn have reduced the subjective need for further information. In other words, this dependent variable may have tapped more into participants’ evaluation of whether they require information than their motivation to act healthily. Empirical evidence strengthens the notion that this variable worked differently than questions about behavioral intentions: It was the only variable *not* directly associated with health attitude (Table 6).

Adding to the impression that information-related questions might be of only limited use as proxies of behavioral spillover is the finding that all participants – irrespective of attitude strength – were less interested in receiving tips about pro-environmental behavior after recalling an environmentally unfriendly (vs. neutral) behavior. Moreover, the predictive power of environmental attitude with respect to interest in pro-environmental tips was also considerably smaller than when other dependent variables were used. The diminished influence of attitude strength suggests that additional processes might be in play when participants make decisions about receiving information.

Also contrary to the prediction that recalling an environmentally unfriendly past behavior would increase pro-environmental tendencies among those with a strong attitude and leave those with a weak attitude unaffected, this condition had no discernible effect among those with a strong attitude, but decreased the support for one pro-environmental petition among participants with a weak attitude. One possible explanation for this pattern is that recalling a past environmentally harmful behavior may have increased the salience of participants’ existing attitude, which then could have led to behavioral patterns consistent with their respective attitude strength. We will discuss these issues in more detail in the next section.

## General Discussion

This research examined whether attitude strength can explain whether the likelihood of engaging in additional behaviors in the domains of environmental protection and health promotion increases (positive spillover) or decreases (negative spillover) after recalling a goal-conducive behavior in the same domain. We argued that when people who have a strong attitude toward an issue carry out a behavior that benefits the issue, such a behavior is an integral part of a wider network of behaviors that serve a more comprehensive, superordinate goal (Carver and Scheier, 2001). We further argued that this mental structure implies that when people with strong attitudes carry out a goal-conducive behavior, it will increase the salience of related behaviors and the importance of continuing to work toward their attitude (or their superordinate goal), not least because failing to do so would elicit cognitive dissonance and negative feelings (Festinger, 1957; Bargh et al., 1992; Ratneshwar et al., 2001; Thøgersen and Crompton, 2009; Thøgersen and Noblet, 2012; Lanzini and Thøgersen, 2014). In short, we predicted that a strong attitude would promote positive spillover and mitigate the risk of negative spillover after an initial goal-conducive behavior (and vice versa: it would promote negative spillover after an initial goal-inconsistent behavior).

Across two studies, we found limited empirical support for the predicted moderating role of attitude strength. In Study 1, attitude strength moderated the effect of a first behavior in two instances: participants with a weak attitude (25th percentile) less strongly intended to act environmentally friendly after recalling an environmentally friendly versus unfriendly action, while participants with a strong attitude (75th percentile) were similarly motivated regardless of the valence of the recalled action. This pattern is consistent with the prediction that a strong attitude toward an issue should promote positive spillover and mitigate the risk of negative spillover after an initial goal-conducive behavior, while those with a weak attitude should feel that they had done enough and not engage in further behaviors in the same behavioral context. A similar pattern was found in Study 2: Recalling an environmentally unfriendly past behavior again had no discernible effect among those with a strong environmental attitude but decreased support for a pro-environmental petition among participants with a weak attitude.

Taken together, these results suggest that a strong attitude can work as a “behavioral stabilizer” that protects against self-complacency and goal disengagement – it keeps people on track. By contrast, a weak attitude can fuel two tendencies that threaten pro-environmental and healthy behavior: First, it can, as suggested by Study 1, make people susceptible to the kind of behavioral fluctuations that are described in the literature as “moral licensing” (Merritt et al., 2010) or the tendency to “rest on one’s laurels” (Amir and Ariely, 2008). Second, a weak attitude can, as suggested by Study 2, increase the susceptibility to disengage entirely from environmental or health goals after an initial setback (i.e., the recall of a goal-inconsistent behavior), a tendency that has been referred to as the “what-the-hell effect” (Cochran and Tesser, 1996; see also Dolan and Galizzi, 2015).

A possible explanation for why participants with a weak environmental attitude acted in line with “moral licensing” (inconsistent behavior or negative spillover) in Study 1 but in line with the “what-the-hell effect” (consistently goal-inconsistent behavior or positive spillover) in Study 2 is that the two samples differed in terms of absolute attitude strength. To examine whether environmental attitude differed across studies, we pooled participants from both studies and recalibrated the Rasch scale (including all items from both studies), so that attitude scores were on the same metric and directly comparable. Participants in Study 1 were more environmentally friendly (*M* = 0.06, *SD* = 0.77) than participants in Study 2 [*M* = -0.91, *SD* = 0.73; *t*(663.84) = 20.87, *p* < 0.001]. Because we defined attitude strength *relative* to other participants in the respective samples, participants with a weak environmental attitude in Study 2 were less environmentally friendly in absolute terms than participants with a weak attitude in Study 1. In other words, participants with a weak attitude in Study 1 probably still cared at least somewhat about the environment and might therefore have displayed the kinds of self-regulation processes well known from research on moral licensing (e.g., Merritt et al., 2010; Jordan et al., 2011; Mullen and Monin, 2016). By contrast, participants with a weak attitude in Study 2 might have felt indifferent or even hostile toward the idea of environmental protection. Recalling an environmentally unfriendly behavior could therefore have highlighted the latter group’s anti-environmental attitude and motivated them to engage in further attitude-consistent behaviors, accounting for the observed consistency in their behavior.

In addition to some interaction effects, this research also found compelling evidence for a direct effect of attitude: Across two studies and in both domains, a stronger attitude was associated with an increased likelihood of engaging in corresponding goal-conducive behaviors. In short, in the context of behavioral spillover, attitude strength assumed two roles – that of a direct predictor and that of a moderator. The direct effect was much more consistent across different dependent variables and contexts than the moderator effect.

In sum, this research provides limited evidence for the idea that attitude strength (as one possible operationalization of relatively stable individual differences in how relevant an issue is to a person) can moderate the extent to which engaging in pro-environmental or healthy behaviors leads to positive or negative spillover.

This finding has implications for theory and practice. First, it provides limited empirical support for plausible but rarely tested assumptions about the role of attitude strength (and similar concepts tapping into personal relevance) in the context of spillover (for notable exceptions, see Effron et al., 2009; Meijers, 2014). As such, our findings improve the field’s understanding for *whom* engaging in a goal-conducive behavior leads to positive or negative spillover.

The findings also contribute to a refined theoretical understanding of the conditions under which recalling past behavior affects subsequent behaviors. Based on Bem’s (1972) self-perception theory, various spillover researchers have argued that reminding people of past goal-consistent behavior (e.g., pro-environmental actions) could lead to or make salient a corresponding identity and thereby increase the tendency to engage in positive spillover (Van der Werff et al., 2014b; Lacasse, 2015, 2016; Truelove et al., 2016). This line of reasoning points to a relatively malleable conceptualization of identity that is best understood as a *mediator* between recalled and subsequent behavior (Van der Werff et al., 2014a,b). Our findings complement this view by suggesting that when conceptualized and measured as traits, identity – and other similar conceptualizations of relatively stable individual differences such as attitude, superordinate goal, or values – can influence how thinking about past behaviors affects spillover. People who have a firm identity or who hold a very favorable or unfavorable attitude about an issue have few doubts about who they are and what they appreciate. It is therefore unlikely that reminders about what they did or failed to do in the past influence how they see themselves, nor should such reminders have much effect on subsequent behaviors. By contrast – and consistent with Bem’s (1972) proposition that people use their behavior to infer information about themselves only “to the extent that internal cues are weak, ambiguous, or uninterpretable” (p. 2) – those with a less firm identity or attitude may find diagnostic value in reminders of past behavior, and adjust subsequent behavior accordingly.

The findings also have implications for practice. It can be assumed that reminding people of past pro-environmental or healthy behaviors (Van der Werff et al., 2014a,b) or labeling them as “environmentalists” or “health-conscious” (Cornelissen et al., 2007; Lacasse, 2016) is an effective strategy to increase positive spillover (after an initial goal-conducive behavior) among those with moderate attitude levels. However, using the same approach is bound to be less effective among those with a firm attitude or identity. A better understanding of how different levels of attitude strength affect spillover can also help campaigners use their resources more efficiently. For instance, our findings suggest that people with a strong attitude are unlikely to display negative spillover. Thus, when trying to reduce negative spillover effects, campaign designers could economize by focusing their efforts on people with moderate and weak attitudes.

A limitation of the research is that attitude strength accounted for positive and negative spillover for only some of the dependent variables. This raises two major questions. First, why did attitude strength moderate the effect of recalling a goal-consistent versus a goal-inconsistent behavior for some but not for other variables? Previous research suggests that when the second behavior is either extremely difficult or extremely easy, it could attenuate or even override the generally positive relationship between attitude strength and the likelihood of engaging in further goal-conducive behaviors (Kaiser and Schultz, 2009; see also Truelove et al., 2014). If this explanation is valid, the anticipated moderating effect of attitude strength should be more likely for intentions that are neither extremely difficult nor easy. However, if the popularity of the dependent variables (see the arithmetic means in Tables 1, 4) is an indication of their difficulty (Kaiser et al., 2007), it can be seen that there is no systematic relationship between item difficulty and whether attitude strength moderated the effect of the recalled behavior. This suggests that the effect of attitude strength on spillover probably did not depend on the difficulty or costs of the behaviors.

On a more speculative note, the fact that the expected moderation was found for only some of the dependent variables could also have to do with the subjective meaning that participants attributed to the respective behaviors. For example, it is possible that participants may have perceived the behaviors as environmentally relevant to different extents (Truelove and Gillis, 2018), and that those with a strong attitude were most likely to engage in behaviors they perceived as impactful. To test this explanation, future research could assess the perceived environmental impact of different behaviors for each participant and examine whether this additional information can help to understand when attitude strength works as a moderator.

The second major question is why did we not find any of the predicted attitude moderations in the health domain. It is striking that much spillover research focuses directly or indirectly on morality, for example, by examining the extent to which engaging in morally relevant behaviors affects people’s self-perceptions and subsequent behaviors (Merritt et al., 2010; Jordan et al., 2011; Mullen and Monin, 2016). A possible mechanism through which morality could affect spillover is by highlighting the violation of personal norms after goal-inconsistent behaviors. That is, the stronger people’s moral norm regarding the relevant behavior, the more would behaving inconsistently induce cognitive dissonance and threaten their self-perception as a moral person. Thus, people with strong moral norms are likely to behave consistently with their norms and goals and thereby avoid these negative cognitions (Thøgersen, 2004).

This raises the question to what extent moral processes are relevant for the two domains examined here. There is evidence that people understand behaviors that affect the environment to be morally relevant (Stern, 2000; Feinberg and Willer, 2013; Van der Werff et al., 2013; Jia et al., 2017), but the extent to which the same applies to caring for one’s own health is less clear. Whereas environmentally harmful actions can negatively affect both the natural environment and other people, eating unhealthily or failing to exercise do not have immediately obvious negative consequences for others, and therefore lack a critical quality of prototypical moral violations (Rottman et al., 2015). It therefore seems plausible that people perceive environmental behavior as more morally charged than health behavior (the comparisons of self-assessed morality of the recalled behaviors support this line of reasoning, see Supplementary Tables 3, 4). In short, to the extent that moral processes play a key role in behavioral spillover, it is possible that such effects – and the corresponding moderation by attitude strength – are more likely to occur in the context of environmental behavior. Future research could test this possibility by comparing the extent to which moral processes are triggered when people engage in environmental versus health behaviors.

One last critical point is that we used several dependent variables, which increased the probability to detect (interaction) effects that do not in fact exist (false positives). This research is exploratory in the sense that it is one of the first to investigate the role of attitude as a moderator of spillover effects and does therefore not necessarily require statistical procedures to correct for false positives (Rothman, 1990; Rubin, 2017). However, to be able to assess the extent to which the rate of false positives might challenge our findings, we used the false discovery rate method (FDR; Benjamini and Hochberg, 1995) to adjust the *p*-values of the interaction terms (i.e., the focal interest of this paper).^5^ Applying the FDR method shifted the two relevant interactions of Study 1 just beyond the 5% significance level (*p*s = 0.056); the two relevant interactions of Study 2 were no longer statistically significant (*p*s ≥ 0.18). Thus, while the FDR adjustments do not completely challenge our findings, they further qualify the already limited moderating effect of attitude strength.

## Conclusion

Overall, the two studies showed that the importance of an issue to a person – in our study operationalized as behavior-based attitude (Kaiser et al., 2007, 2010) – had a direct and positive effect on decisions and behaviors. Additionally, we found limited evidence for the prediction that a strong (favorable) attitude increases the consistency of goal-conducive behavior, whereas a weak attitude was associated with less predictable behavioral patterns. This lends some support to the theoretical considerations derived from goal-theoretical perspectives and self-perception theory (for more details, see Höchli et al., 2018). The findings are relevant for theory because they point to a possible boundary condition of positive and negative spillover. Practically they matter because they enable those seeking to effect change to more accurately anticipate the effects of campaigns and interventions on different groups of people, which should help to allocate resources more efficiently and render campaigns more effective.

## Data Availability Statement

The raw data and R code supporting the conclusions of this manuscript is available in the Open Science Framework https://osf.io/mbgxh/.

## Ethics Statement

At the time these studies were conducted (spring 2013 and summer 2018), our faculty had no Internal Review Board to grant ethical approval. However, we certify that the research adhered to the ethical principles of the American Psychological Association [APA] (2010). Informed consent was attained by asking participants to continue only if they were willing to participate and if they had read and understood the instructions and information provided. Participants were told that participation was voluntary and that they had the right to withdraw from the study at any time. Upon completion of the study, participants were fully debriefed. The data were anonymized and treated confidentially.

## Author Contributions

AB conceived and designed Study 1, analyzed the data, and wrote the first draft of the manuscript. BH and AB conceived, designed, and analyzed the data from Study 2. BH contributed to the editing process of the first draft and added additional content. Both authors contributed to manuscript revision, and read and approved the submitted version.

## Conflict of Interest Statement

The authors declare that the research was conducted in the absence of any commercial or financial relationships that could be construed as a potential conflict of interest.

## Footnotes

1 Importantly, though, the Campbell paradigm does not suggest that a single behavior can be equated with attitude. The latent trait can be inferred only by inspecting a broad range of behaviors, ordered by difficulty.

2 The Johnson-Neyman technique suggested that the recall condition would also have an effect on participants with an extremely favorable environmental attitude (i.e., scores larger than 4.08). However, because our sample did not include any participants with such extreme scores, this extrapolated effect should be seen as hypothetical and treated with caution.

3 change.org

4 thepetitionsite.com

5 Note that limiting the FDR adjustment to the interaction terms results in their *p*-values being larger as compared to when the *p*s of all predictors are corrected.

## References

[B1] Albarracín D. Wyer R. S. (2000). The cognitive impact of past behavior: influences on beliefs, attitudes, and future behavioral decisions. *J. Pers. Soc. Psychol.* 79 5–22. 10.1037/0022-3514.79.1.5 10909874 PMC4807731

[B2] American Psychological Association [APA] (2010). *Ethical Principles of Psychologists and Code of Conduct.* Retrieved from American Psychological Association Available at: http://www.apa.org/ethics/code/principles.pdf

[B3] AmirO.ArielyD. (2008). Resting on laurels: the effects of discrete progress markers as subgoals on task performance and preferences. *J. Exp. Psychol. Learn. Mem. Cogn.* 34 1158–1171. 10.1037/a0012857 18763898

[B4] Bamberg S. Möser G. (2007). Twenty years after Hines, Hungerford, and Tomera: a new meta-analysis of psycho-social determinants of pro-environmental behaviour. *J. Environ. Psychol.* 27 14–25. 10.1016/j.jenvp.2006.12.002

[B5] Bargh J. A. Chaiken S. Govender R. Pratto F. (1992). The generality of the automatic attitude activation effect. *J. Pers. Soc. Psychol.* 62 893–912. 10.1037//0022-3514.62.6.893 1619549

[B6] Bem D. J. (1972). Self-perception theory. *Adv. Exp. Soc. Psychol.* 6 1–62.

[B7] Benjamini Y. Hochberg Y. (1995). Controlling the false discovery rate: a practical and powerful approach to multiple testing. *J. R. Stat. Soc. Ser. B Methodol.* 57 289–300. 10.1111/j.2517-6161.1995.tb02031.x

[B8] Blanken I. van de Ven N. Zeelenberg M. (2015). A meta-analytic review of moral licensing. *Pers. Soc. Psychol. Bull.* 41 540–558. 10.1177/0146167215572134 25716992

[B9] Boekaerts M. de Koning E. Vedder P. (2006). Goal-directed behavior and contextual factors in the classroom: an innovative approach to the study of multiple goals. *Educ. Psychol.* 41 33–51. 10.1207/s15326985ep4101_5

[B10] Bond T. G. Fox C. M. (2007). *Applying the Rasch Model: Fundamental Measurement in the Human Sciences.* Mahwah, NJ: Lawrence Erlbaum.

[B11] Bratt C. (1999). Consumers’ environmental behavior: generalized, sector-based, or compensatory? *Environ. Behav.* 31 28–44. 10.1177/00139169921971985

[B12] Brügger A. Dorn M. H. Messner C. Kaiser F. G. (2019). Conformity within the Campbell paradigm: the proposition of a new measurement instrument. *Soc. Psychol.* (in press).

[B13] Brügger A. Kaiser F. G. Roczen N. (2011). One for all? Connectedness to nature, inclusion of nature, environmental identity, and implicit association with nature. *Eur. Psychol.* 16 324–333. 10.1027/1016-9040/a000032

[B14] Byrka K. Kaiser F. G. (2013). Health performance of individuals within the Campbell paradigm. *Int. J. Psychol.* 48 986–999. 10.1080/00207594.2012.702215 22857604

[B15] Byrka K. Kaminska K. (2015). Can recycling compensate for speeding on highways? Similarity and difficulty of behaviors as key characteristics of green compensatory beliefs. *Pol. Psychol. Bull.* 46 477–487. 10.1515/ppb-2015-0054

[B16] Campbell D. T. (1963). “Social attitudes and other acquired behavioral dispositions,” in *Psychology: A Study of a Science*, ed. Koch S. (New York, NY: McGraw-Hill), 94–172. 10.1037/10590-003

[B17] Carrico A. R. Raimi K. T. Truelove H. B. Eby B. (2018). Putting your money where your mouth is: an experimental test of pro-environmental spillover from reducing meat consumption to monetary donations. *Environ. Behav.* 50 723–748. 10.1177/0013916517713067

[B18] Carver C. S. Scheier M. F. (2001). *On the Self-Regulation of Behavior.* Cambridge: Cambridge University Press.

[B19] Cochran W. Tesser A. (1996). “The ‘what the hell’ effect: some effects of goal proximity and goal framing on performance,” in *Striving and Feeling: Interactions among Goals, Affect, and Self-Regulation*, eds Martin L. L. Tesser A. (Hillsdale, NJ: Lawrence Erlbaum Associates, Inc),99–120.

[B20] Cohen J. Cohen P. West S. G. Aiken L. S. (2003). *Applied Multiple Regression/Correlation analysis for the Behavioral Sciences*, 3rd Edn Mahwah, NJ: Lawrence Erlbaum.

[B21] Conway P. Peetz J. (2012). When does feeling moral actually make you a better person? Conceptual abstraction moderates whether past moral deeds motivate consistency or compensatory behavior. *Pers. Soc. Psychol. Bull.* 38 907–919. 10.1177/0146167212442394 22492550

[B22] Cornelissen G. Bashshur M. R. Rode J. Le Menestrel M. (2013). Rules or consequences? The role of ethical mind-sets in moral dynamics. *Psychol. Sci.* 24 482–488. 10.1177/0956797612457376 23447556

[B23] Cornelissen G. Dewitte S. Warlop L. Yzerbyt V. (2007). Whatever people say I am, that’s what I am: social labeling as a social marketing tool. *Int. J. Res. Mark.* 24 278–288. 10.1016/j.ijresmar.2007.05.001 22165427

[B24] Crowne D. P. Marlowe D. (1960). A new scale of social desirability independent of psychopathology. *J. Consult. Psychol.* 24 349–354. 10.1037/h0047358 13813058

[B25] Dillman D. A. (2001). *Mail and Internet Surveys: The Tailored Design Method.* New York, NY: Wiley.

[B26] Dolan P. Galizzi M. M. (2015). Like ripples on a pond: behavioral spillovers and their implications for research and policy. *J. Econ. Psychol.* 47 1–16. 10.1016/j.joep.2014.12.003

[B27] Dunlap R. E. Van Liere K. D. (1978). The new environmental paradigm. *J. Environ. Educ.* 9 10–19. 10.1080/00958964.1978.10801875

[B28] Effron D. A. Cameron J. S. Monin B. (2009). Endorsing Obama licenses favoring Whites. *J. Exp. Soc. Psychol.* 45 590–593. 10.1016/j.jesp.2009.02.001

[B29] Faul F. Erdfelder E. Buchner A. Lang A.-G. (2009). Statistical power analyses using G^∗^Power 3.1: tests for correlation and regression analyses. *Behav. Res. Methods* 41 1149–1160. 10.3758/BRM.41.4.1149 19897823

[B30] Feinberg M. Willer R. (2013). The moral roots of environmental attitudes. *Psychol. Sci.* 24 56–62. 10.1177/0956797612449177 23228937

[B31] Festinger L. (1957). *A theory of Cognitive Dissonance.* Stanford, CA: Stanford University Press.

[B32] Fishbach A. Dhar R. Zhang Y. (2006). Subgoals as substitutes or complements: the role of goal accessibility. *J. Pers. Soc. Psychol.* 91 232–242. 10.1037/0022-3514.91.2.232 16881761

[B33] Freedman J. L. Fraser S. C. (1966). Compliance without pressure: the foot-in-the-door technique. *J. Pers. Soc. Psychol.* 4 195–202. 10.1037/h0023552 5969145

[B34] Gatersleben B. Murtagh N. Abrahamse W. (2014). Values, identity and pro-environmental behaviour. *Contemp. Soc. Sci.* 9 374–392. 10.1080/21582041.2012.682086

[B35] Gawronski B. Strack F. (2012). *Cognitive Consistency: A Fundamental Principle in Social Cognition.* New York, NY: Guilford.

[B36] Godin G. Kok G. (1996). The Theory of Planned Behavior: a review of its applications to health-related behaviors. *Am. J. Health Promot.* 11 87–98. 10.4278/0890-1171-11.2.87 10163601

[B37] Hagger M. S. Anderson M. Kyriakaki M. Darkings S. (2007). Aspects of identity and their influence on intentional behavior: comparing effects for three health behaviors. *Pers. Individ. Dif.* 42 355–367. 10.1016/j.paid.2006.07.017

[B38] Hines J. M. Hungerford H. R. Tomera A. N. (1986). Analysis and synthesis of research on responsible environmental behavior: a meta-analysis. *J. Environ. Educ.* 18 1–8. 10.1080/00958964.1987.9943482

[B39] Höchli B. Brügger A. Messner C. (2018). How focusing on superordinate goals motivates broad, long-term goal pursuit: a theoretical perspective. *Front. Psychol.* 9:1879. 10.3389/fpsyg.2018.01879 30333781 PMC6176065

[B40] Hoyle R. H. Sherrill M. R. (2006). Future orientation in the self-system: possible selves, self-regulation, and behavior. *J. Pers.* 74 1673–1696. 10.1111/j.1467-6494.2006.00424.x 17083662

[B41] Jia F. Soucie K. Alisat S. Curtin D. Pratt M. (2017). Are environmental issues moral issues? Moral identity in relation to protecting the natural world. *J. Environ. Psychol.* 52 104–113. 10.1016/j.jenvp.2017.06.004 29071291

[B42] Jordan J. Mullen E. Murnighan J. K. (2011). Striving for the moral self: the effects of recalling past moral actions on future moral behavior. *Pers. Soc. Psychol. Bull.* 37 701–713. 10.1177/0146167211400208 21402752

[B43] Kaiser F. G. Brügger A. Hartig T. Bogner F. X. Gutscher H. (2014). Appreciation of nature and appreciation of environmental protection: how stable are these attitudes and which comes first? *Rev. Eur. Psychol. Appl.* 64 269–277. 10.1016/j.erap.2014.09.001

[B44] Kaiser F. G. Byrka K. Hartig T. (2010). Reviving Campbell’s paradigm for attitude research. *Pers. Soc. Psychol. Rev.* 14 351–367. 10.1177/1088868310366452 20435803

[B45] Kaiser F. G. Hartig T. Brügger A. Duvier C. (2013). Environmental protection and nature as distinct attitudinal objects: an application of the Campbell paradigm. *Environ. Behav.* 45 369–398. 10.1177/0013916511422444

[B46] Kaiser F. G. Kibbe A. Arnold O. (2017). “Self-determined, enduring, ecologically sustainable ways of life: attitude as a measure of individuals’ intrinsic motivation,” in *Handbook of Environmental Psychology and Quality of Life Research*, eds Fleury-Bahi G. Pol E. Navarro O. (Cham: Springer International Publishing), 185–195. 10.1007/978-3-319-31416-7_10

[B47] Kaiser F. G. Oerke B. Bogner F. X. (2007). Behavior-based environmental attitude: development of an instrument for adolescents. *J. Environ. Psychol.* 27 242–251. 10.1016/j.jenvp.2007.06.004

[B48] Kaiser F. G. Schultz P. W. (2009). The attitude–behavior relationship: a test of three models of the moderating role of behavioral difficulty. *J. Appl. Soc. Psychol.* 39 186–207. 10.1111/j.1559-1816.2008.00435.x

[B49] Kaiser F. G. Wilson M. (2004). Goal-directed conservation behavior: the specific composition of a general performance. *Pers. Individ. Dif.* 36 1531–1544. 10.1016/j.paid.2003.06.003

[B50] Kaklamanou D. Jones C. R. Webb T. L. Walker S. R. (2015). Using public transport can make up for flying abroad on holiday: compensatory green beliefs and environmentally significant behavior. *Environ. Behav.* 47 184–204. 10.1177/0013916513488784

[B51] Karp D. G. (1996). Values and their effect on pro-environmental behavior. *Environ. Behav.* 28 111–133. 10.1177/0013916596281006

[B52] Kashima Y. Paladino A. Margetts E. A. (2014). Environmentalist identity and environmental striving. *J. Environ. Psychol.* 38 64–75. 10.1016/j.jenvp.2013.12.014

[B53] Kibbe A. (2011). *Gesundheitseinstellung im Rahmen des Campbell-Paradigmas: Entwicklung Eines Verhaltensbasierten Messinstrumentes.* Magdeburg: Otto-von-Guericke-Universität.

[B54] Kortenkamp K. V. Moore C. F. (2006). Time, uncertainty, and individual differences in decisions to cooperate in resource dilemmas. *Pers. Soc. Psychol. Bull.* 32 603–615. 10.1177/0146167205284006 16702154

[B55] Kruglanski A. W. Shah J. Y. Fishbach A. Friedman R. Chun W. Y. Sleeth-Keppler D. (2002). A theory of goal systems. *Adv. Exp. Soc. Psychol.* 34 331–378.

[B56] Lacasse K. (2015). The importance of being green: the influence of green behaviors on Americans’ political attitudes toward climate change. *Environ. Behav.* 47 754–781. 10.1177/0013916513520491 24826365

[B57] Lacasse K. (2016). Don’t be satisfied, identify! Strengthening positive spillover by connecting pro-environmental behaviors to an “environmentalist” label. *J. Environ. Psychol.* 48 149–158. 10.1016/j.jenvp.2016.09.006

[B58] Lanzini P. Thøgersen J. (2014). Behavioural spillover in the environmental domain: an intervention study. *J. Environ. Psychol.* 40 381–390. 10.1016/j.jenvp.2014.09.006

[B59] Long J. A. (2018). *jtools: Analysis and Presentation of Social Scientific Data.* Available at: https://cran.r-project.org/package=jtools (accessed 4 8 2019).

[B60] Longoni C. Gollwitzer P. M. Oettingen G. (2014). A green paradox: validating green choices has ironic effects on behavior, cognition, and perception. *J. Exp. Soc. Psychol.* 50 158–165. 10.1016/j.jesp.2013.09.010

[B61] Margetts E. A. Kashima Y. (2017). Spillover between pro-environmental behaviours: the role of resources and perceived similarity. *J. Environ. Psychol.* 49 30–42. 10.1016/j.jenvp.2016.07.005

[B62] Masuda A. D. Kane T. D. Shoptaugh C. F. Minor K. A. (2010). The role of a vivid and challenging personal vision in goal hierarchies. *J. Psychol.* 144 221–242. 10.1080/00223980903472235 20461929

[B63] Mazar N. Zhong C.-B. (2010). Do green products make us better people? *Psychol. Sci.* 21 494–498. 10.1177/0956797610363538 20424089

[B64] Meijers M. H. C. (2014). *On Justifying Eco-Unfriendly Behaviors.* Amsterdam: University of Amsterdam.

[B65] Meijers M. H. C. Noordewier M. K. Avramova Y. R. (2014). “I just recycled. Can I use the car now? When people continue or discontinue behaving sustainably after an initial sustainable act,” in *Encouraging Sustainable Behavior: Psychology and the Environment*, ed. van Trijp H. C. M. (New York, NY: Psychology Press), 71–80.

[B66] Meijers M. H. C. Verlegh P. W. J. Noordewier M. K. Smit E. G. (2015). The dark side of donating: how donating may license environmentally unfriendly behavior. *Soc. Influ.* 10 250–263. 10.1080/15534510.2015.1092468

[B67] Merritt A. C. Effron D. A. Monin B. (2010). Moral self-licensing: when being good frees us to be bad. *Soc. Pers. Psychol. Compass* 4 344–357. 10.1111/j.1751-9004.2010.00263.x

[B68] Milfont T. L. (2009). The effects of social desirability on self-reported environmental attitudes and ecological behaviour. *Environmentalist* 29 263–269. 10.1007/s10669-008-9192-2

[B69] Monin B. Miller D. T. (2001). Moral credentials and the expression of prejudice. *J. Pers. Soc. Psychol.* 81 33–43. 10.1177/0956797610363538 11474723

[B70] Moskowitz G. B. (2012). “The representation and regulation of goals,” in *Goal-Directed Behavior*, eds Aarts H. Elliot A. J. (New York, NY: Psychology Press), 1–47.

[B71] Mullen E. Monin B. (2016). Consistency versus licensing effects of past moral behavior. *Annu. Rev. Psychol.* 67 363–385. 10.1146/annurev-psych-010213-115120 26393870

[B72] Nigbur D. Lyons E. Uzzell D. (2010). Attitudes, norms, identity and environmental behaviour: using an expanded theory of planned behaviour to predict participation in a kerbside recycling programme. *Br. J. Soc. Psychol.* 49 259–284. 10.1348/014466609X449395 19486547

[B73] Nilsson A. Bergquist M. Schultz W. P. (2017). Spillover effects in environmental behaviors, across time and context: a review and research agenda. *Environ. Educ. Res.* 23 573–589. 10.1080/13504622.2016.1250148

[B74] Noblet C. L. McCoy S. K. (2018). Does one good turn deserve another? Evidence of domain-specific licensing in energy behavior. *Environ. Behav.* 50 839–863. 10.1177/0013916517718022

[B75] Ogunbode C. A. Henn L. Tausch N. (2018). Context-appropriate environmental attitude measurement in Nigeria using the Campbell paradigm. *Environ. Dev. Sustain* 1–8. 10.1007/s10668-018-0281-1

[B76] Olli E. Grendstad G. Wollebaek D. (2001). Correlates of environmental behaviors: bringing back social context. *Environ. Behav.* 33 181–208. 10.1177/0013916501332002

[B77] Otto S. Kröhne U. Richter D. (2018). The dominance of introspective measures and what this implies: the example of environmental attitude. *PLoS One* 13:e0192907. 10.1371/journal.pone.0192907 29447235 PMC5814007

[B78] Otto S. Pensini P. (2017). Nature-based environmental education of children: environmental knowledge and connectedness to nature, together, are related to ecological behaviour. *Glob. Environ. Change* 47 88–94. 10.1016/j.gloenvcha.2017.09.009

[B79] Oyserman D. James L. (2011). “Possible identities,” in *Handbook of Identity Theory and Research*, eds Schwartz S. J. Luyckx K. Vignoles V. L. (New York, NY: Springer), 117–145. 10.1007/978-1-4419-7988-9_6

[B80] Ratneshwar S. Barsalou L. W. Pechmann C. Moore M. (2001). Goal-derived categories: the role of personal and situational goals in category representations. *J. Consum. Psychol.* 10 147–157. 10.1207/s15327663jcp1003_3

[B81] Reips U.-D. (2002). Standards for Internet-based experimenting. *Exp. Psychol.* 49 243–256. 10.1026//1618-3169.49.4.243 12455331

[B82] Rothman K. J. (1990). No adjustments are needed for multiple comparisons. *Epidemiology* 1 43–46. 10.1097/00001648-199001000-00010 2081237

[B83] Rottman J. Kelemen D. Young L. (2015). Hindering harm and preserving purity: how can moral psychology save the planet? *Philos. Compass* 10 134–144. 10.1111/phc3.12195

[B84] Rubin M. (2017). Do p values lose their meaning in exploratory analyses? It depends how you define the familywise error rate. *Rev. Gen. Psychol.* 21 269–275. 10.1037/gpr0000123

[B85] Sachdeva S. Iliev R. Medin D. L. (2009). Sinning saints and saintly sinners: the paradox of moral self-regulation. *Psychol. Sci.* 20 523–528. 10.1111/j.1467-9280.2009.02326.x 19320857

[B86] Schultz P. W. (2001). The structure of environmental concern: concern for self, other people, and the biosphere. *J. Environ. Psychol.* 21 327–339. 10.1006/jevp.2001.0227

[B87] Schultz P. W. Gouveia V. V. Cameron L. D. Tankha G. Schmuck P. Franěk M. (2005). Values and their relationship to environmental concern and conservation behavior. *J. Cross Cult. Psychol.* 36 457–475. 10.1177/0022022105275962

[B88] Schwartz S. H. (1992). Universals in the content and structure of values: theoretical advances and empirical tests in 20 countries. *Adv. Exp. Soc. Psychol.* 25 1–65. 10.1016/s0065-2601(08)60281-6 17874285

[B89] Schwartz S. H. Melech G. Lehmann A. Burgess S. Harris M. Owens V. (2001). Extending the cross-cultural validity of the theory of basic human values with a different method of measurement. *J. Cross Cult. Psychol.* 32 519–542. 10.1177/0022022101032005001

[B90] Shah J. Y. Kruglanski A. W. (2003). When opportunity knocks: bottom-up priming of goals by means and its effects on self-regulation. *J. Pers. Soc. Psychol.* 84 1109–1122. 10.1037/0022-3514.84.6.1109 12793579

[B91] Sintov N. Geislar S. White L. V. (2019). Cognitive accessibility as a new factor in proenvironmental spillover: results from a field study of household food waste management. *Environ. Behav.* 51 50–80. 10.1177/0013916517735638

[B92] Smolders K. C. H. J. de Kort Y. A. W. Tenner A. D. Kaiser F. G. (2012). Need for recovery in offices: behavior-based assessment. *J. Environ. Psychol.* 32 126–134. 10.1016/j.jenvp.2011.12.003

[B93] Sparks P. Guthrie C. A. (1998). Self-identity and the Theory of Planned Behavior: a useful addition or an unhelpful artifice? *J. Appl. Soc. Psychol.* 28 1393–1410. 10.1111/j.1559-1816.1998.tb01683.x

[B94] Spiller S. A. Fitzsimons G. J. Lynch J. G. McClelland G. H. (2013). Spotlights, floodlights, and the magic number zero: simple effects tests in moderated regression. *J. Mark. Res.* 50 277–288. 10.1509/jmr.12.0420

[B95] Steinhorst J. Klöckner C. A. Matthies E. (2015). Saving electricity – For the money or the environment? Risks of limiting pro-environmental spillover when using monetary framing. *J. Environ. Psychol.* 43 125–135. 10.1016/j.jenvp.2015.05.012

[B96] Stern P. C. (2000). Toward a coherent theory of environmentally significant behavior. *J. Soc. Issues* 56 407–424. 10.1111/0022-4537.00175

[B97] Stern P. C. Dietz T. Guagnano G. A. (1998). A brief inventory of values. *Educ. Psychol. Meas.* 58 984–1001. 10.1177/0013164498058006008

[B98] Theodorakis Y. (1994). Planned behavior, attitude strength, role identity, and the prediction of exercise behavior. *Sport Psychol.* 8 149–165. 10.1123/tsp.8.2.149

[B99] Thøgersen J. (2004). A cognitive dissonance interpretation of consistencies and inconsistencies in environmentally responsible behavior. *J. Environ. Psychol.* 24 93–103. 10.1016/s0272-4944(03)00039-2

[B100] Thøgersen J. Crompton T. (2009). Simple and painless? The limitations of spillover in environmental campaigning. *J. Consum. Policy* 32 141–163. 10.1007/s10603-009-9101-1

[B101] Thøgersen J. Noblet C. (2012). Does green consumerism increase the acceptance of wind power? *Energy Policy* 51 854–862. 10.1016/j.enpol.2012.09.044

[B102] Thøgersen J. Ölander F. (2006). To what degree are environmentally beneficial choices reflective of a general conservation stance? *Environ. Behav.* 38 550–569. 10.1177/0013916505283832

[B103] Truelove H. B. Carrico A. R. Weber E. U. Raimi K. T. Vandenbergh M. P. (2014). Positive and negative spillover of pro-environmental behavior: an integrative review and theoretical framework. *Glob. Environ. Change* 29 127–138. 10.1016/j.gloenvcha.2014.09.004

[B104] Truelove H. B. Gillis A. J. (2018). Perception of pro-environmental behavior. *Glob. Environ. Change* 49 175–185. 10.1016/j.gloenvcha.2018.02.009

[B105] Truelove H. B. Yeung K. L. Carrico A. R. Gillis A. J. Raimi K. T. (2016). From plastic bottle recycling to policy support: an experimental test of pro-environmental spillover. *J. Environ. Psychol.* 46 55–66. 10.1016/j.jenvp.2016.03.004

[B106] Urban J. (2016). Are we measuring concern about global climate change correctly? Testing a novel measurement approach with the data from 28 countries. *Clim. Change* 139 397–411. 10.1007/s10584-016-1812-0

[B107] Vallacher R. R. Wegner D. M. (1987). What do people think they’re doing? Action identification and human behavior. *Psychol. Rev.* 94 3–15. 10.1037/0033-295X.94.1.3

[B108] Van der Werff E. Steg L. Keizer K. (2013). It is a moral issue: the relationship between environmental self-identity, obligation-based intrinsic motivation and pro-environmental behaviour. *Glob. Environ. Change* 23 1258–1265. 10.1016/j.gloenvcha.2013.07.018

[B109] Van der Werff E. Steg L. Keizer K. (2014a). Follow the signal: when past pro-environmental actions signal who you are. *J. Environ. Psychol.* 40 273–282. 10.1016/j.jenvp.2014.07.004

[B110] Van der Werff E. Steg L. Keizer K. (2014b). I am what I am, by looking past the present: the influence of biospheric values and past behavior on environmental self-identity. *Environ. Behav.* 46 626–657. 10.1177/0013916512475209

[B111] Weber E. U. (1997a). “Perception and expectation of climate change: precondition for economic and technological adaptation,” in *Environment, Ethics, and Behavior: The Psychology of Environmental Valuation and Degradation The New Lexington Press Management series and the New Lexington Press Social and Behavioral Science Series*, eds Bazerman M. H. Messick D. M. Tenbrunsel A. E. Wade-Benzoni K. A. (San Francisco, CA: The New Lexington Press), 314–341.

[B112] Weber E. U. (1997b). “Perception and expectation of climate change: precondition for economic and technological adaptation,” in *Psychological Perspectives to Environmental and Ethical Issues in Management*, eds Bazerman M. H. Messick D. M. Tensbrunsel A. Wade-Benzoni K. (San Francisco, CA: Jossey-Bass), 314–341.

[B113] Weibel C. Messner C. Brügger A. (2014). Completed egoism and intended altruism boost healthy food choices. *Appetite* 77 38–45. 10.1016/j.appet.2014.02.010 24576466

[B114] Whitmarsh L. O’Neill S. (2010). Green identity, green living? The role of pro-environmental self-identity in determining consistency across diverse pro-environmental behaviours. *J. Environ. Psychol.* 30 305–314. 10.1016/j.jenvp.2010.01.003

[B115] Wittenbrink B. Schwarz N. (2007). *Implicit Measures of Attitudes.* New York, NY: Guilford Press.

