# Corrigendum: The Role of Attitude Strength in Behavioral Spillover: Attitude Matters—But Not Necessarily as a Moderator

**DOI:** 10.3389/fpsyg.2019.02990

**Published:** 2020-01-24

**Authors:** Adrian Brügger, Bettina Höchli

**Affiliations:** Department of Consumer Behavior, Faculty of Business, Economics and Social Sciences, University of Bern, Bern, Switzerland

**Keywords:** pro-environmental behavior, health behavior, environmental attitude, health attitude, spillover, moral licensing, moral cleansing

In the original article, there was an error. The **Data Availability** statement was inadvertently omitted during the production process, and should be as follows:

“The raw data and R code supporting the conclusions of this manuscript is available in the Open Science Framework https://osf.io/mbgxh/.”

The authors apologize for this error and state that this does not change the scientific conclusions of the article in any way. The original article has been updated.

