# Supplementary material for: The Role of Attitude Strength in Behavioral Spillover: Attitude Matters—But Not Necessarily as a Moderator
Source: Front Psychol. 2019 May 9;10:1018. doi: 10.3389/fpsyg.2019.01018 (PMC6520604; doi:10.3389/fpsyg.2019.01018)
Supplement: Supplementary file 1 [file Data_Sheet_1.docx]

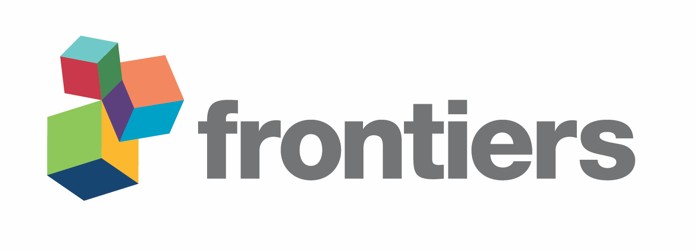


***Supplementary Material***

The Role of Attitude Strength in Behavioral Spillover: Attitude Matters–But Not Necessarily as a Moderator

# Adrian Brügger*, Bettina Höchli

*** Correspondence:** Corresponding Author: [adrian.bruegger@imu.unibe.ch](mailto:adrian.bruegger@imu.unibe.ch)


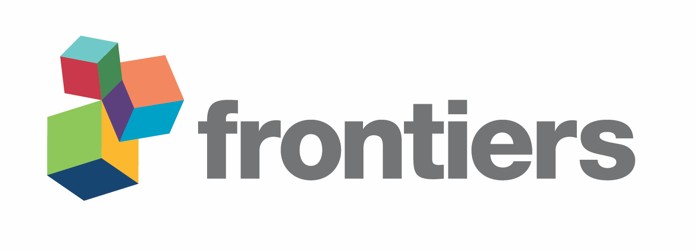


# Supplementary Table 1.

## Behavior-based environmental attitude items.

| Item | English | German | Format | Study 1 | Study 2 | Ref. |
| --- | --- | --- | --- | --- | --- | --- |
| 1 | After a picnic, I leave the place as clean as it was | Ich verlasse nach einem Picknick den Platz genauso, wie |  |  |  |  |
|  | originally | ich ihn angetroffen habe | D | x | x | 1 |
| *2* | *At red traffic lights, I keep the engine running* | *Vor roten Ampeln lasse ich den Motor laufen* | *F* | *x* | *x* | *1* |
| *3* | *For longer journeys (more than 6 h), I take an airplane* | *Für längere Reisen (6 Stunden und länger) nehme ich das* |  |  |  |  |
|  |  | *Flugzeug* | *F* | *x* | *x* | *1* |
| *4* | *I drive my car in or into the city* | *Ich fahre mit dem Auto in die Stadt bzw. ich fahre in der* |  |  |  |  |
|  |  | *Stadt Auto* | *F* | *x* | *x* | *1* |
| 5 | I am a member of a car pool | Ich bin in einem CarSharing-Pool | D | x | x | 1 |
| 6 | I am a member of an environmental organization | Ich bin Mitglied in einer Umweltschutzorganisation | D | x | x | 1 |
| 7 | I boycott companies with an unecological background | Ich boykottiere Produkte von Firmen, die sich nachweislich |  |  |  |  |
|  |  | umweltschädigend verhalten | F | x | x | 1 |
| 8 | I bring empty bottles to a recycling bin | Altglas bringe ich zum Sammelcontainer | F | x | x | 1 |
| *9* | *I buy beverages in cans* | *Ich kaufe Getränke in Dosen* | *F* | *x* | *x* | *1* |
| *10* | *I buy bleached and colored toilet paper* | *Ich kaufe gebleichtes und gefärbtes Toilettenpapier* | *F* | *x* | *x* | *1* |
| *11* | *I buy convenience foods* | *Ich kaufe Fertiggerichte* | *F* | *x* | *x* | *1* |
| 12 | I buy domestically grown wooden furniture | Ich kaufe Möbel aus einheimischen Hölzern | F | x | x | 1 |
| 13 | I buy meat and produce with eco-labels | Ich kaufe Lebensmittel aus kontrolliert biologischem Anbau | F | x | x | 1 |
| 14 | I buy products in refillable packages | Ich kaufe Artikel in Nachfüllpackungen | F | x | x | 1 |
| 15 | I buy seasonal produce | Ich kaufe Obst und Gemüse der Jahreszeit entsprechend | F | x | x | 1 |
| 16 | I collect and recycle used paper | Ich sammle altes Papier und gebe es zum Recycling | F | x | x | 1 |
| 17 | I contribute financially to environmental organizations | Ich spende Geld für Umweltschutzorganisationen | F | x | x | 1 |
| 18 | I drive in such a way as to keep my fuel consumption as | Durch mein Fahrverhalten versuche ich, den |  |  |  |  |
|  | low as possible | Kraftstoffverbrauch so niedrig wie möglich zu halten | D | x | x | 1 |
| 19 | I drive on freeways at speeds under 100 kph (= 62.5 | Ich fahre auf der Autobahn höchstens 100 km/h |  |  |  |  |
|  | mph) |  | F | x | x | 1 |
| *20* | *I drive to where I want to start my hikes* | *Zum Spazierengehen fahre ich mit dem Auto an den* |  |  |  |  |
| 21 | I have looked into the pros and cons having a private | *Ausgangspunkt des Spazierganges*  Ich habe mich über Vor- und Nachteile einer Solaranlage | *F* | *x* | *x* | *1* |
|  | source of solar power | informiert | D | x | x | 1 |
| 22 | I have pointed out unecological behavior to someone | Ich mache jemanden, der / die sich umweltschädigend | F | x | x | 1 |

|  |  | verhält, darauf aufmerksam |  |  |  |  |
| --- | --- | --- | --- | --- | --- | --- |
| *23* | *I keep the engine running while waiting in front of a* | *Vor geschlossenen Bahnschranken lasse ich den Motor* |  |  |  |  |
|  | *railroad crossing or in a traffic jam* | *laufen* | *F* | *x* | *x* | *1* |
| *24* | *I kill insects with a chemical insecticide* | *Insekten bekämpfe ich mit chemischen Mitteln* | *F* | *x* | *x* | *1* |
| 25 | I own a fuel-efficient automobile (less than 7 l per 100 | Ich besitze ein verbrauchsreduziertes Auto (weniger als 7 |  |  |  |  |
|  | km; i.e., less than 3 gallons per 100 miles) | Liter Treibstoff pro 100 km) | D | x | x | 1 |
| *26* | *I put dead batteries in the garbage* | *Leere Batterien werfe ich in den Hausmüll* | *D* | *x* | *x* | *1* |
| 27 | I refrain from owning a car | Ich verzichte auf ein Auto | D | x | x | 1 |
| 28 | I reuse my shopping bags | Ich verwende Einkaufstüten oder -taschen mehrfach | D | x | x | 1 |
| 29 | I ride a bicycle or take public transportation to work or | Für den Arbeits- bzw. Schulweg benutze ich das Fahrrad, |  |  |  |  |
|  | school | öffentliche Verkehrsmittel oder gehe zu Fuss | F | x | x | 1 |
| *30* | *I use a chemical air freshener in my bathroom* | *In der Toilette benutze ich chemische Duftsteine für den* |  |  |  |  |
|  |  | *guten Geruch* | *D* | *x* | *x* | *1* |
| *31* | *I use a clothes dryer* | *Ich benutze einen Wäschetrockner* | *F* | *x* | *x* | *1* |
| *32* | *I use fabric softener with my laundry* | *Ich benutze beim Waschen einen Weichspüler* | *D* | *x* | *x* | *1* |
| 33 | I wait until I have a full load before doing my laundry | Ich warte, bis ich eine volle Wäschetrommel habe, bevor |  |  |  |  |
|  |  | ich wasche | F | x | x | 1 |
| 34 | I wash dirty clothes without prewashing | Beim Waschen verzichte ich auf den Vorwaschgang | F | x | x | 1 |
| *35* | *If I am offered a plastic bag in a store, I take it* | *Wenn ich in einem Geschäft eine Plastiktüte bekomme,* |  |  |  |  |
|  |  | *nehme ich sie* | *F* | *x* | *x* | *1* |
| *36* | *In hotels, I have the towels changed daily* | *Im Hotel lasse ich täglich die Handtücher wechseln* | *D* | *x* | *x* | *1* |
| *37* | *In the winter, I keep the heat on so that I do not have to* | *In meiner Wohnung ist es im Winter so warm, dass man* |  |  |  |  |
|  | *wear a sweater* | *ohne Pullover nicht friert* | *D* | *x* | *x* | *1* |
| *38* | *In the winter, I leave the windows open for long periods* | *Um zu lüften, lasse ich auch im Winter das Fenster längere* |  |  |  |  |
|  | *of time to let in fresh air* | *Zeit offen* | *F* | *x* | *x* | *1* |
| 39 | In winter, I turn down the heat when I leave my apartment for more than 4 hours | Im Winter drehe ich meine Heizung herunter, wenn ich meine Wohnung für mehr als 4 Stunden verlasse | F | x | x | 1 |
| *40* | *After meals, I dispose of leftovers in the toilet* | *Breiige Essensreste leere ich in die Toilette* | *D* | *x* | - | *1* |
| 41 | I bought solar panels to produce energy | Ich habe eine Solaranlage zur Energieerzeugung |  |  |  |  |
|  |  | angeschafft | D | x | - | 1 |
| 42 | I buy milk in returnable bottles | Ich kaufe die Milch in der Mehrwegflasche | F | x | - | 1 |
| 43 | I own energy-efficient household devices | Ich benutze verbrauchsarme Haushaltsgeräte | D | x | - | 1 |
| 44 | I prefer to shower rather than to take a bath | Ich bevorzuge es, zu duschen statt zu baden | F | x | - | 1 |
| 45 | I get books and other materials that are concerned with environmental problems | Ich besorge mir Bücher, Informationsschriften oder andere Materialien, die sich mit Umweltproblemen befassen | F | x | - | 1 |
| 46 | I requested an estimate on having solar power installed | Ich habe Angebote zur Anschaffung einer Solaranlage |  |  |  |  |
|  |  | eingeholt | D | x | - | 1 |
| 47 | I talk with friends about problems related to the | Ich unterhalte mich mit Bekannten über Probleme der |  |  |  |  |
| environment Umweltverschmutzung F | | | | x | - | 1 |

Supplementary Material

| *48* | *I use an oven cleaning spray to clean my oven* | *Zum Reinigen des Backofens verwende ich ein Spray* | *F* | *x* | - | *1* |
| --- | --- | --- | --- | --- | --- | --- |
| 49 | I use renewable energy sources | Ich nutze erneuerbare Energiequellen zur Stromerzeugung | D | x | - | 1 |
| 50 | In nearby areas (around 30 km; around 20 miles), I use | Für Fahrten in die umliegende Gegend (bis 30 km) benütze |  |  |  |  |
|  | public transportation or ride a bike | ich öffentliche Nahverkehrsmittel oder das Fahrrad | F | x | - | 1 |
| 51 | I read about environmental issues | Ich lese Artikel zu Umweltfragen | F | - | x | 1 |
| 52 | I am a vegetarian | Ich bin Vegetarier/in | D | - | x | 2 |
| 53 | I have a contract for renewable energy with my energy provider | Ich habe einen Vertrag für erneuerbare Energien mit meinem Stromanbieter | D | - | x | 2 |
| 54 | I own an energy efficient dishwasher (efficiency class | Ich besitze eine energieeffiziente Geschirrspülmaschine |  |  |  |  |
|  | A+ or better) | (Effizienzklasse A+ oder besser) | D | - | x | 2 |
| 55 | I own solar panels | Ich besitze eine Solaranlage | D | - | x | 2 |
| 56 | I shower (rather than taking a bath) | Ich dusche (statt zu baden) | F | - | x | 2 |
| 57 | I buy beverages and other liquids in returnable bottles | Ich kaufe Getränke und andere Flüssigkeiten in |  |  |  |  |
|  |  | Mehrwegflaschen | F | - | x | 2 |
| 58 | I talk with friends about environmental pollution, | Ich spreche mit Freunden über Umweltverschmutzung, |  |  |  |  |
|  | climate change, and/or energy consumption | Klimawandel und/oder Energieverbrauch | F | - | x | 2 |

*Note*. Items in italics = negatively formulated behaviors recoded prior to analysis D = items presented in dichotomous (yes/no) format

F = items presented in 5-point frequency format and then dichotomized (see text) x = items used in respective study

1 = Kaiser and Wilson (2004); 2 = new items made available by Florian G. Kaiser.

**Supplementary Table 2.**

***Behavior-based health attitude items.***

| Item English German Format | Study 1 | Study 2 | Ref. |
| --- | --- | --- | --- |
| 1 At least 15 minutes a day, I take time to go for a walk Ich gehe mindestens 15 Minuten täglich spazieren D | x | x | 1 |
| 2 At least twice a day, I brush my teeth Ich putze mir mindestens zweimal täglich die Zähne D | x | x | 1 |
| 1. At least twice a week, I floss my teeth Ich benutze mindestens zweimal wöchentlich Zahnseide D 2. At least twice a year, I have my teeth checked Ich gehe mindestens zwei Mal im Jahr zur   Zahnvorsorgeuntersuchung D | x  x | x  x | 1  1 |
| *5 I allow pets in the kitchen Ich dulde Haustiere in meiner Küche D* | *x* | *x* | 1 |
| 6 I am a member of a sport facility or club Ich bin Mitglied in einem Sportverein/ Fitnessstudio D | x | x | 1 |
| 7 I avoid eating salty foods or adding salt to my food Ich vermeide salzige Speisen D | x | x | 1 |
| 8 I avoid fast food Ich vermeide Fastfood D/F | x | x | 1 |
| 1. I avoid sweets Ich vermeide Süssigkeiten D 2. I check the consumption / best-before dates of food Ich überprüfe das Haltbarkeitsdatum von Nahrungsmitteln   products F | x  x | x  x | 1  1 |
| 11 I clean cans before opening them Ich reinige Konservendosen bevor ich sie öffne F | x | x | 1 |
| 12 I count calories Ich zähle Kalorien D | x | x | 1 |
| 1. *I cross streets on a red light Ich überquere bei Rot die Strasse F* 2. *I drink more than a glass of wine or a beer a day Ich trinke täglich mehr als ein Glas Wein oder ein kleines*   *Bier D* | *x*  *x* | *x*  *x* | 1  1 |
| *15 I eat after 21.00 hrs. / 9 pm Ich esse nach 21 Uhr F* | *x* | *x* | 1 |
| 16 I eat fruits or vegetables daily Ich esse täglich Obst und Gemüse D | x | x | 1 |
| 17 I exercise at least 15 minutes per day Ich treibe mindestens 15 Minuten täglich Sport D | x | x | 1 |
| *18 I get drunk Ich betrinke mich F* | *x* | *x* | 1 |
| 19 I go for one-day hikes Ich mache Tageswanderungen F | x | x | 1 |
| 20 I go to bed before 22.00 hrs. / 10 pm Ich gehe vor 22 Uhr ins Bett F | x | x | 1 |
| 21 I have a hobby Ich habe ein Hobby D | x | x | 1 |
| 1. I have purchased sports gear Ich besitze Sportgeräte/ -kleidung/ -ausrüstung D 2. I meditate or practice yoga Ich praktiziere Entspannungstechniken (Yoga, Meditation,   o.ä.) F | x  x | x  x | 1  1 |
| 24 I practice sports regularly (swimming, football, etc.) Ich trainiere regelmässig (Fussball, Schwimmen, o.ä.) D | x | x | 1 |

Supplementary Material

1. I regularly examine myself for cancer Ich untersuche mich selbst regelmässig auf Anzeichen von

Krebs

| D | x | x | 1 |
| --- | --- | --- | --- |
| F/D | x | x | 1 |
| *D/F* | *x* | *x* | 1 |
| F | x | x | 1 |
| F | x | x | 1 |
| F | x | x | 1 |
| F | x | x | 1 |
| D | x | x | 1 |
| F | x | x | 1 |
| D/F | x | x | 1 |
| F | x | x | 1 |
| *F* | *x* | *x* | 1 |
| *F* | *x* | *x* | 1 |
| F | x | x | 1 |
| *F* | *x* | *x* | 1 |
| D | x | x | 1 |
| F | x | x | 1 |
| F | x | x | 1 |
| F | x | x | 1 |
| D | x | - | 1 |
| D | x | - | 1 |
| F | x | - | 1 |
| F | x | - | 2 |
| F | x | - | 2 |
| F | x | - | 2 |
| F | x | - | 2 |
| D | x | - | 2 |

1. I sleep at least 7 hours per night Ich schlafe mindestens 7 Stunden pro Nacht
2. *I smoke Ich rauche*
3. I spend time in nature Ich verbringe Zeit an der frischen Luft
4. I spend time with other people to socialize Ich verbringe Zeit damit, meine sozialen Kontakte zu

pflegen

1. I take my breaks at work Ich mache während der Arbeit ausreichend Pausen
2. I take time to relax Ich nehme mir Zeit, mich zu entspannen
3. I use sunscreen Ich benutze Sonnencreme
4. I wash dishes right after a meal or at least on the same day

Ich spüle mein benutztes Geschirr noch am gleichen Tag

1. I wash fruits and vegetables Ich esse nur gewaschenes Obst und Gemüse
2. I wash meat before preparing it Ich wasche Fleisch vor der Zubereitung
3. *I work overtime Ich leiste Überstunden*
4. *I would rather take the elevator than the stairs Ich benutze eher den Aufzug als die Treppe*
5. In cars, I wear seatbelts Ich schnalle mich im Auto an
6. *In the evening or during weekends, I work at home Ich arbeite zusätzlich abends oder am Wochenende zuhause*
7. My meals last at least 10 minutes Ich nehme mir zum Essen mindestens 10 Minuten Zeit
8. When it is cold, I wear warm clothes Wenn es kalt ist, ziehe ich mich warm an
9. With food, I read the description of content Ich lese Nährwerttabellen von Lebensmitteln
10. With new medication, I read the package insert Ich lese vor dem ersten Gebrauch den Beipackzettel von

Medikamenten

1. I keep an exercise diary Ich führe ein Trainingstagebuch
2. I possess a fitness video Ich besitze ein Fitnessvideo/ DVD mit Fitnessübungen
3. To let in fresh air, I open windows for a long period of time

Ich lüfte meine Wohnung ausgiebig

1. I wash my hands before I cook or eat Ich wasche meine Hände vor dem Kochen oder Essen
2. I use wellness offers (e.g. sauna, massage) Ich nutze Wellnessangebote (zB Sauna, Massage)
3. I protect myself from sexually transmitted diseases (e.g. through condoms)

Ich schütze mich vor sexuell übertragbaren Krankheiten (zB durch Kondome)

1. I wear a helmet when cycling Ich trage beim Fahrradfahren einen Helm
2. I drink at least 2 litres per day Ich trinke mindestens 2 Liter pro Tag

| 52 | After using a restroom, I wash my hands | Ich wasche mir nach dem Toilettengang die Hände |
| --- | --- | --- |
| 53 | I wake up at the same time every day | Ich stehe jeden Tag zur gleichen Zeit auf |
| 54 | I clean my smartphone to destroy bacteria | Ich reinige mein Smartphone, um Bakterien abzutöten |
| 55 | I have fitness wearables | Ich besitze Fitness-Wearables |
| *56* | *I leave the dirty dishes until the next morning* | *Ich lasse schmutziges Geschirr bis zum nächsten Morgen* |
| 57 | I regularly disinfect my keyboard | *zurück*  Ich desinfiziere meine Tastatur regelmäßig |
| *58* | *I regularly eat in front of a computer* | *Ich esse regelmäßig vor einem Computer* |
| 59 | I regularly stand up while working at a desk | Ich stehe regelmäßig auf, während ich an einem Schreibtisch |
|  |  | arbeite |
| *60* | *I text while driving* | *Ich schreibe während dem Autofahren SMS* |
| 61 | I use an app to keep track of my exercise | Ich benutze eine App, um den Überblick über mein Training |
|  |  | zu behalten |

| F | - x | 1 |
| --- | --- | --- |
| D | - x | 3 |
| F | - x | 3 |
| D | - x | 3 |
| *F* | - *x* | 3 |
| D | - x | 3 |
| *D* | - *x* | 3 |
| F | - x | 3 |
| *F* | - *x* | 3 |
| D | - x | 3 |

*Note.* Items in italics = negatively formulated behaviors recoded prior to the analysis D = items presented in dichotomous (yes/no) format

F = items presented in a 5-point frequency format and then dichotomized. The presence of both D and F codes indicates that different response formats were used in Study 1 and Study 2.

x = item used in respective study

1 = Byrka and Kaiser (2013), 2 = Kibbe (2011), 3 = newly developed items.


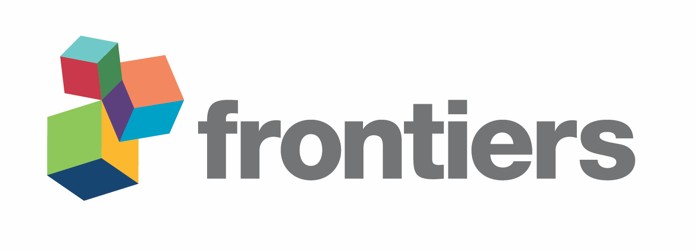


**Supplementary Table 3.**

***Fixed-effects ANOVA results using self-assessed morality as the criterion, Study 2.***

Sum

Mean

2 90% CI

Predictor of

*df* Square *F p* partial η

[LL, UL]

Squares

| (Intercept) 287.83 | 1 | 287.83 | 217.73 | .000 |  | |
| --- | --- | --- | --- | --- | --- | --- |
| Recall 793.27 | 4 | 198.32 | 150.02 | .000 | .39 | [.35, .43] |
| Error 1220.16 | 923 | 1.32 |  |  |  |  |

condition

**Supplementary Table 4.**

***Tukey HSD comparisons of self-assessed morality between the experimental recall conditions, Study 2.***

TUKEY HSD

| Group | Mean | *SD* | Env pos | Env neg | Health pos | Health neg |
| --- | --- | --- | --- | --- | --- | --- |
| Environment Positive | 1.85 | 1.17 |  |  |  |  |
| Environment  Negative  Health Positive | -0.71  0.98 | 0.96  1.35 | <.001  <.001 <.001 | | | |
| Health Negative | -0.09 | 0.83 | <.001 <.001 <.001 | | | |
| Control | 1.24 | 1.34 | <.001 <.001 .20 <.001 | | | |
